# Cerium Methacrylate Assisted Preparation of Highly Thermally Conductive and Anticorrosive Multifunctional Coatings for Heat Conduction Metals Protection

**DOI:** 10.1007/s40820-023-01163-w

**Published:** 2023-08-18

**Authors:** Fei Xu, Peng Ye, Jianwen Peng, Haolei Geng, Yexiang Cui, Di Bao, Renjie Lu, Hongyu Zhu, Yanji Zhu, Huaiyuan Wang

**Affiliations:** 1 School of Chemical Engineering and Technology, Tianjin University, Tianjin, 300350 People’s Republic of China; 2 Haihe Laboratory of Sustainable Chemical Transformations, Tianjin, 300192 People’s Republic of China; 3 Tianjin Key Lab Composite & Functional Materials, School of Materials Science and Engineering, Tianjin University, Tianjin, 300072 People’s Republic of China; 4 State Key Laboratory of Chemical Engineering and Collaborative Innovation Center of Chemical Science and Engineering (Tianjin), Tianjin University, Tianjin, 300072 People’s Republic of China; 5 Tianjin Key Laboratory of Chemical Process Safety and Equipment Technology, Tianjin University, Tianjin, 300072 People’s Republic of China

**Keywords:** Epoxy coatings, Thermal conductivity, Anti-corrosion, Hydrophobicity, Cerium methacrylate

## Abstract

Preparing polymeric coatings with well corrosion resistance and high thermal conductivity (TC) to prolong operational life and ensure service reliability of heat conductive metallic materials has long been a substantive and urgent need while a difficult task. Here we report a multifunctional epoxy composite coating (F-CB/CEP) by synthesizing cerium methacrylate and ingeniously using it as a novel curing agent with corrosion inhibit for epoxy resin and modifier for boron nitride through "cation-π" interaction. The prepared F-CB/CEP coating presents a high TC of 4.29 W m^−1^ K^−1^, which is much higher than other reported anti-corrosion polymer coatings and thereby endowing metal materials coated by this coating with outstanding thermal management performance compared with those coated by pure epoxy coating. Meanwhile, the low-frequency impedance remains at 5.1 × 10^11^ Ω cm^2^ even after 181 days of immersion in 3.5 wt% NaCl solution. Besides, the coating also exhibits well hydrophobicity, self-cleaning properties, temperature resistance and adhesion. This work provides valuable insights for the preparation of high-performance composite coatings with potential to be used as advanced multifunctional thermal management materials, especially for heat conduction metals protection.

**Supplementary Information:**

The online version contains supplementary material available at 10.1007/s40820-023-01163-w.

## Highlights

- Cerium methacrylate was prepared and ingeniously used as curing agent for epoxy, surface modifier for BN, and effective corrosion inhibitor.
- The coating integrates high thermal conductivity (4.29 W m^−1^ K^−1^), corrosion resistance (low-frequency impedance is 5.1 × 10^11^ Ω cm^2^ at 181 day), hydrophobicity (WCA: ~ 148.35° and WSA: ~ 20°), temperature resistance and adhesion.
- The coating can extend service life of metal materials without sacrificing their inherent heat conduction property.

**Supplementary Information:**

The online version contains supplementary material available at 10.1007/s40820-023-01163-w.

## Graphical abstract

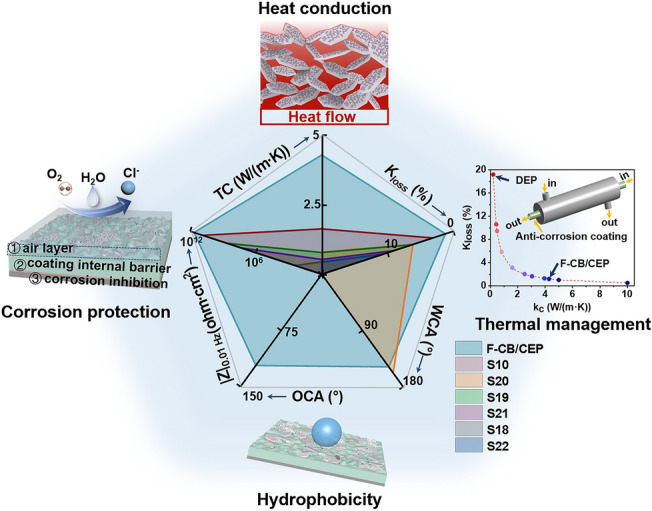

## Introduction

Along with technology development of high power, miniaturization and energy saving, the accompanying heat transfer and heat accumulation problems have led to urgent demands for advanced and effective thermal management materials [1–3]. Most metals possess satisfactory thermal conductivity (TC), making them favorable candidates for heat dissipation materials [4, 5]. Nonetheless, they are extremely susceptible to aggressive and corrosive environment, causing serious deterioration problems of metal structure and properties being responsible for important economic and security burdens [6, 7]. Recently, intensive work has been devoted to applying polymer-based composite coatings with well adhesion on metal substate, denseness, and ease of processing for protecting metal materials by isolating corrosive medium [8]. Moreover, due to the air cushion between coating and liquid could act as effective physical barrier to prevent corrosive medium from directly wetting the coating surface, hydrophobic surfaces with well water-repellency have also aroused lots of interests in reducing corrosion behaviors of metal materials [9]. Hence, polymer composite coating with hydrophobic surface, combining the multiple advantages of polymer matrix, anti-corrosion filler, and hydrophobic properties is considered to be potential candidate for metal protection.

In spite of numerous efforts have been attempted to employ composite coatings to successfully protect metals from corrosion, the accompanying reduction in thermal management capacity of the coated metals is usually ignored with little published literature [10]. TC of most anti-corrosion polymer coatings has been stuck in a relatively low level (< 0.5 W m^−1^ K^−1^), 2–3 orders of magnitude lower than that of metallic materials. As reported in our previous work [11], for a specific shell-and-tube heat exchanger, if pure epoxy coating with thickness of 110 μm and TC of 0.22 W m^−1^ K^−1^ is applied to the outside of the heat exchange tube, the overall heat transfer coefficient of the coated heat exchanger will be lost by 9.47% compared with the pristine heat exchanger. This would cause substantially extra economic and energy losses during the long-term use. Therefore, high TC of composite coatings is required to maintain the thermal management capability of thermally conductive metallic materials protected by coatings.

Directly adding highly thermally conductive fillers into polymer matrix by physical compounding is an effective method to enhance TC of polymer coatings. Compared with other thermally conductive fillers, intrinsic high TC, excellent electrical insulation, and well physical barrier ability of two-dimensional (2D) boron nitride (BN) makes it attractive to impart both thermally conductive and anticorrosive abilities to the coating [12–14]. Unfortunately, direct addition of inorganic BN with chemical inertness into organic polymer matrix easily causes defects within the composite system due to the poor interfacial compatibility between BN and polymer. This results in increased interfacial thermal resistance (ITR) and rapid penetration of corrosive medium [15, 16]. Generally, modification of BN is beneficial to reduce defects in the organic/inorganic composite system [17]. However, this usually involves complex processes, and intrinsically low TC of modifier always leads to limited TC enhancement of final composites [18, 19]. Therefore, how to break through the bottleneck of improving TC of anticorrosive coatings by simple and effective methods is still an imperative challenge.

It is known that the fatty acid-based metal carboxylates could cure epoxy resins through ring-opening the epoxy groups and forming ion crosslinking network [20]. Moreover, researchers have been dedicated to modifying fillers through "cation-π" interaction between metallic cations and fillers with surface π-electron rich property for enhancing the thermally conductive or anticorrosive performance of composites [21, 22]. Among the numerous metal ions, cerium ion exhibits excellent active inhibition [23, 24]. Meanwhile, the abundant oxygen-containing groups on metal carboxylates make them susceptible to be surface modified by fluorosilane to impart hydrophobicity to the fillers [17]. Thus, it is speculated that cerium carboxylate can be used as both curing agent with corrosion inhibition for epoxy resin and ideal modifier for BN to conveniently and effectively enhance comprehensive performance of the composite coatings.

Herein, to prepare thermally conductive anti-corrosion coating, we synthesized cerium methacrylate (CMA), and ingeniously used it as curing agent for epoxy resin, surface modifier for BN, and corrosion inhibitor. Meanwhile, the CMA-modified BN (CB) was further fluorination modified by fluorosilane. Through these, a unique heterogeneous filler (F-CB), which integrated well heat conduction, corrosion protection, corrosion inhibition and water repellent properties, was prepared. Then the F-CB was filled into epoxy matrix cured by CMA for preparing composite coating (F-CB/CEP) with outstanding comprehensive performance through facile spraying approach. The feasibility of CMA as curing agent for epoxy resin and the formed F-CB with "point-surface" heterostructure were characterized in detail. Then, heat conduction property of the composite coating was investigated and characterized by TC measurement and infrared thermographic technology. Corrosion resistance and corrosion inhibition of the coating and filler were evaluated by electrochemical impedance spectroscopy (EIS) and potentiodynamic polarization measurements. Besides, surface wettability, temperature resistance and adhesion of the coating were also investigated. The unique composite coating, which is simultaneously characterized by high TC, corrosion resistance, hydrophobic performance, temperature resistance and adhesion becomes a promising alternative to traditional anticorrosive coatings.

## Experimental Section

### Materials

Please refer to Text S1 in Supporting Information for details.

### Specimen Preparation

#### Preparation of CMA

Ce(NO_3_)_3_·6H_2_O (7.38 wt%) and NH_4_HCO_3_ (4.03 wt%) were added sequentially into aqueous solution at continuous stirring. The mixture was then stirred at room temperature for 4 h. The resulting mixture was filtered, washed and dried at 80 °C to obtain cerium carbonate (Ce_2_(CO_3_)_3_). CMA was then synthesized through double decomposition reaction between Ce_2_(CO_3_)_3_ (2.13 wt%) and methacrylic acid (MAA, 4.76 wt%) in aqueous solution under continuous stirring at 90 °C. The obtained transparent solution was directly dried at high temperature to obtain CMA.

#### Preparation of CB and F-CB

**Fig. 1:**
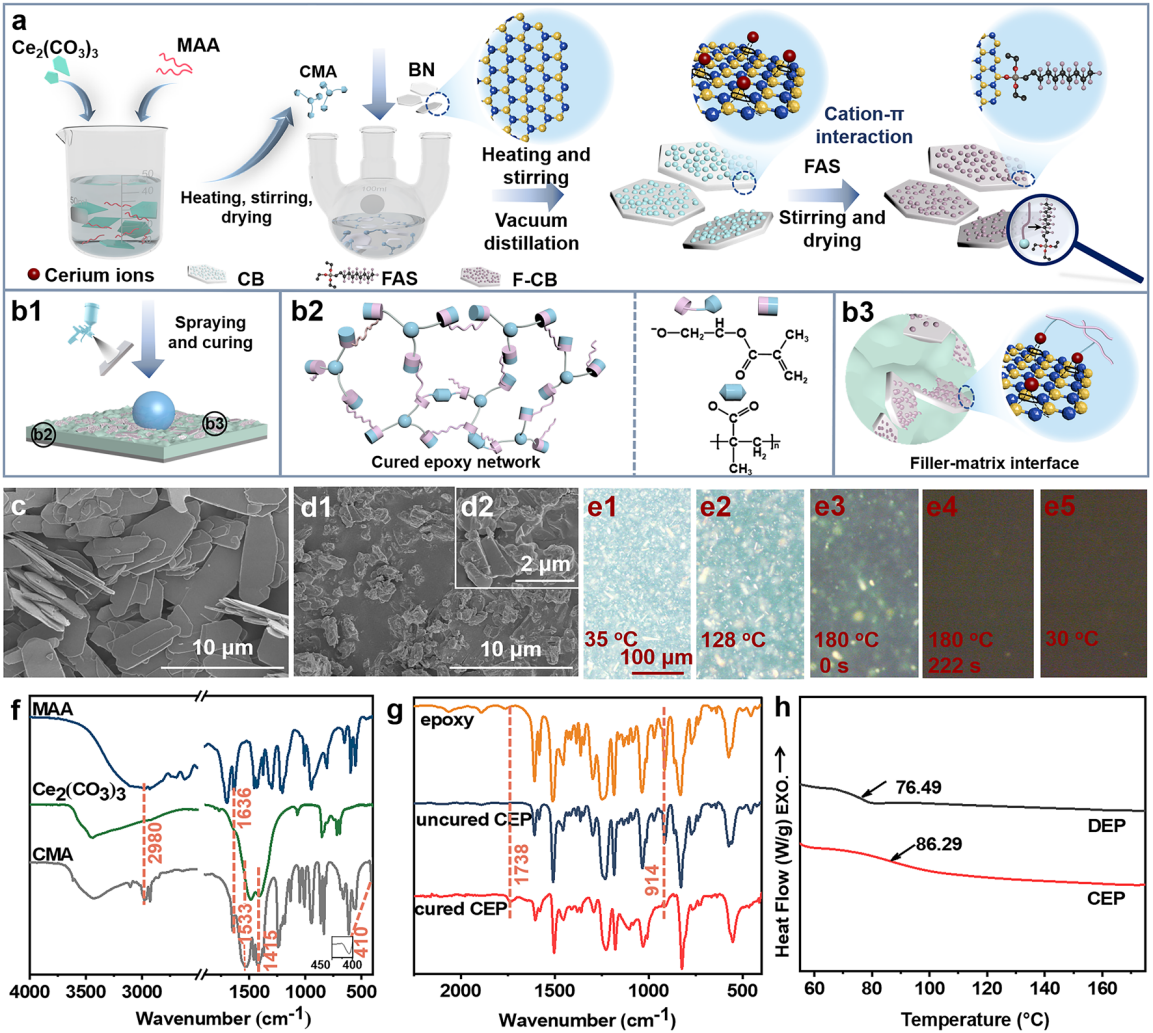
Graphical representation for the fabrication process of **a** CMA and F-CB hybrid filler, and **b1** F-CB/CEP coating. Schematic diagram of **b2** CMA cured epoxy network and **b3** filler-epoxy matrix interface. SEM images of **c** Ce_2_(CO_3_)_3_, **d1, d2** CMA**. e1-e5** Variation of CMA crystals in epoxy matrix with the curing temperature and time captured by polarization microscope. **e1-e5** have the same scale. FT-IR spectra of **f** MAA, Ce_2_(CO_3_)_3_, CMA, as well as **g** epoxy, uncured and cured CEP. **h** DSC curves of DEP and CEP coatings

For the preparation of CB, firstly, CMA (1.03 wt%) was dissolved into deionized water at 80 °C. Subsequently, BN (6.18 wt%) was added into the above solution with further mixing for 3 h. Afterward, CB hybrids were obtained after removing the water through vacuum distillation. F-CB with low surface energy was prepared through modifying CB with 1H, 1H, 2H, 2H-perfluorodecyltriethoxysilane (FAS). Specifically, 400 μL of FAS was dispersed in ethyl acetate by sonication and stirring. Subsequently, 4 g of CB was added into the mixture and the mixture was further stirred and mixed for 7 h. After drying the mixture at 80 °C, the F-CB hybrid filler was obtained with the special ration of FAS, CMA and BN is about 0.91:1:6. The specific preparation process is shown in Fig. 1a.

#### Preparation of the F-CB/CEP Composite Coating

To remove oxide layers and impurities from surface of metal substrates, the substrates were sanded using 400# and 1000# silicon carbide sandpapers in succession. After cleaning in ethanol, the metal substrates were then dried at 80 °C for further use. The F-CB/CEP coating (CMA is used as curing agent) is used as an example to illustrate the preparation process of composite coatings. First, F-CB, epoxy resin, and CMA (mass ratio of CMA to epoxy resin is 1:10) were dispersed in ethyl acetate by continuous stirring and sonication. The prepared dispersion was directly applied to the pre-treated substrate by a simple spraying process. Finally, composite coatings with different BN contents (10–50 wt%, the mass ratio of BN to the total mass of F-CB, epoxy resin, and CMA) were obtained after curing at 140, 160, and 180 °C for 2 h, respectively. For comparison, BN/CEP, BN/DEP, CEP, and DEP coatings were prepared at the similar process. Notably, DEP means that the commercial D230 was used as the curing agent for epoxy resin, and the ratio of D230 to epoxy resin was 1:3. Moreover, the D230-cured coatings were obtained by curing at 40, 90, and 120 °C for 2 h, respectively. Without special emphasis, the content of BN within the composite coatings mentioned below is 50 wt%. The thickness of the coatings is 110 ± 10 μm.

### Characterization

Please refer to the Text S2 for details.

## Results and Discussion

### Synthesis and Characterization of CMA and CEP

The intermediate product Ce_2_(CO_3_)_3_ was first synthesized, and then CMA was synthesized through double decomposition reaction between Ce_2_(CO_3_)_3_ and MAA. As shown in Fig. 1c, d, Ce_2_(CO_3_)_3_ reveals smooth 2D layered structure, and CMA exhibits irregular particles morphology formed by the agglomeration of nanoscale particles. FT-IR spectra were tested to validate the successful synthesis of CMA (Fig. 1f and Text S3). The practicability of CMA as curing agent for epoxy resin was also verified and illustrated in Fig. 1g and Text S4. Polarization microscope was used to explore the variations of CMA crystals during the curing process of epoxy (Fig. 1e). As temperature increased, micron-sized CMA crystals began to melt and move with the flow of epoxy resin. When the temperature stabilized at 180 °C for a period of time, almost all the visible CMA crystals were disappeared. Finally, when it returned to room temperature, the epoxy resin was successfully cured and no recrystallization process occurred to produce new crystals. This further indicates that the melted CMA crystals would involve in the curing of epoxy resin at high temperature. The specific might reactions during the curing of epoxy resin with CMA as curing agent are shown in Fig. S1. CEP coating exhibits a denser crosslinking network as evidenced by the higher glass transition temperature of CEP coating (86.29 °C) compared with that of DEP coating (76.49 °C) as shown in Fig. 1h [25], which is expected to provide better barrier properties to corrosive medium. This is related to the ion crosslinking network-dominated dual-crosslinking networks formed during the curing process as illustrated in Text S4.

### Preparation and Characterization of F-CB Hybrid Filler

**Fig. 2:**
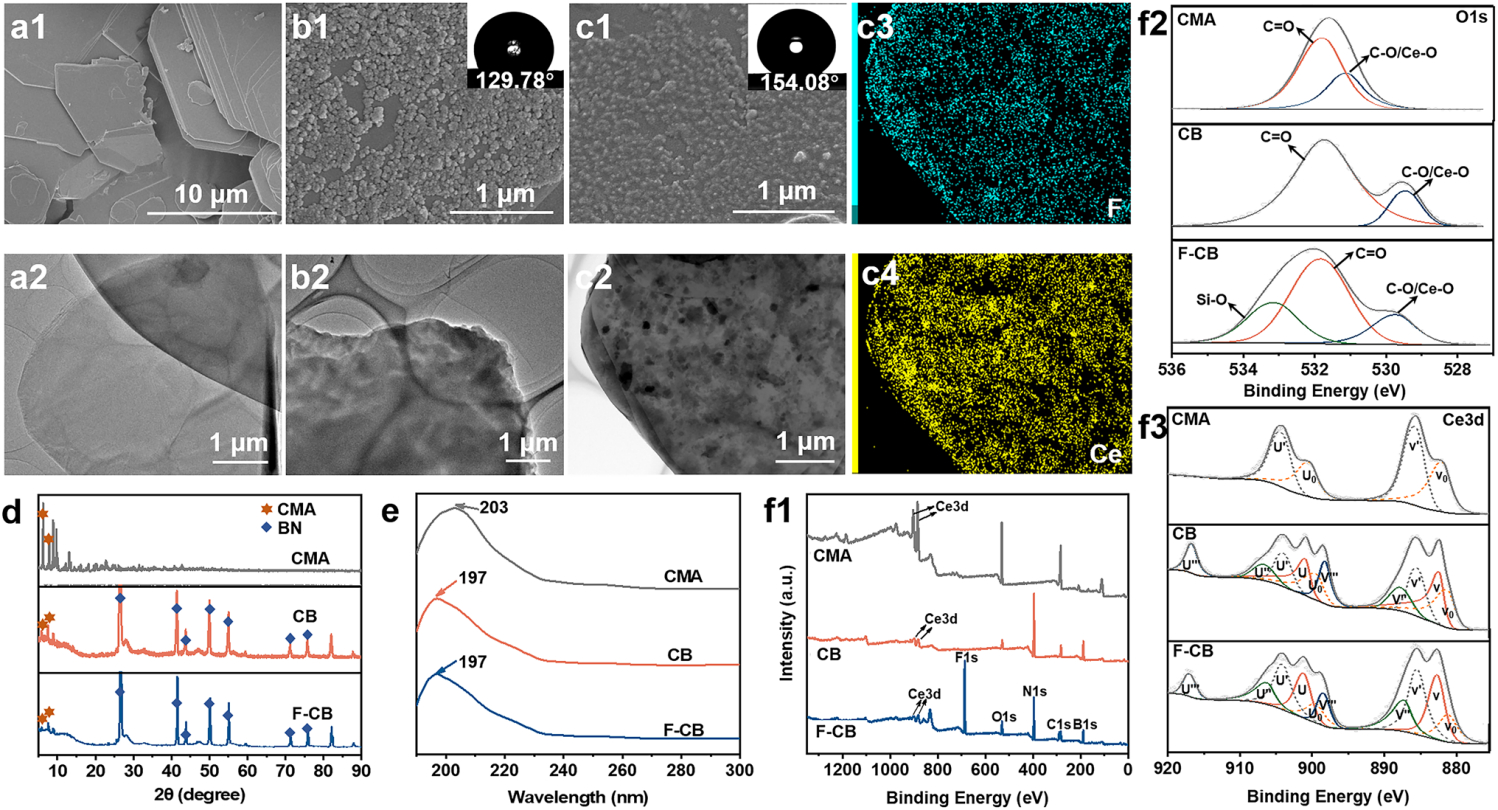
Characterization of the fillers. SEM and TEM images of **a1, a2** BN, **b1, b2** CB, **c1, c2** F-CB, respectively. Insets are the WCA results for the corresponding powders. **c3, c4** Mapping results of **c2** F-CB. **d** XRD patterns, **e** UV–vis spectra, and **f1-f3** XPS results of CMA, CB and F-CB, respectively

During the experiment, we found that CMA can be easily dissolved in deionized water and recrystallized after the complete evaporation of water. It is thought that introduction of BN with surface π-electron rich property during the above recrystallization process could provide active nucleation sites for the recrystallization of CMA due to the "cation-π" interaction between BN and cerium ions [26]. Therefore, we attempted to modify BN with CMA as modifier. Original BN exhibits a smooth 2D sheet-like morphology (Fig. 2a). As expected, Fig. 2b shows that CB exhibits a perfect "point-surface" heterogeneous structure with the recrystallized cerium methacrylate hybrids (named as h-CMAs, the reason for naming them hybrids is illustrated in Text S6) grow uniformly as nanoparticles on the surface of 2D BN. After further modification of CB with FAS, h-CMAs still disperse on BN surface in the form of nanoparticles (Fig. 2c1, c2), while graininess of the nanoparticles is not as obvious as before fluoridation. This is related to the homogeneous coverage of FAS on CB surface during the hydrolysis condensation process. The uniform and dense dispersion of F and Ce elements in the mapping results in Fig. 2c3, c4 indicates that BN was successfully modified by CMA and FAS. And the water contact angle (WCA) of F-CB raises to 154.08° ± 4.01° (insets in Fig. 2b1, c1), which is beneficial to endow the final coating with effective water resistance. XRD characterization further demonstrates the successful modification of BN as well as the crystal structure of the fillers (see details in Text S5). UV–vis absorbance spectroscopy was performed to prove the interaction between BN and h-CMAs on its surface (Fig. 2e). Compared with that of CMA (203 nm), the characteristic absorption of CB (197 nm) and F-CB (197 nm) all show clear blue shift, which is related to the "cation-π" interaction between cerium ions and BN [27]. XPS survey was conducted to analyze the specific valence of cerium ions during the preparation process for filler and the interaction between cerium ions and BN in detail (Fig. 2f and Text S6).

### SEM images of the Composite Coatings

It is believed that the filler dispersion state plays an important role in deciding the performance of the polymer composites [28]. Therefore, SEM of different coatings were investigated and shown in Figs. S2, S3 and Text S7. For F-CB/CEP coating (Fig. S2e), both the surface and cross-sectional SEM images show that the filler form well interconnected network and no obvious defects inside the coating. This is attributed to the enhanced interfacial compatibility between BN and epoxy matrix due to the well bridging effect provided by h-CMAs nanoparticles on BN surface, that is, cationic end is attached to BN surface and methacrylate end might be involved in the curing of epoxy resin. These facilitate the rapid heat transfer and well impermeability of the coating. Meanwhile, the "point-surface" heterogeneous structures of uniformly dispersed h-CMAs nanoparticles on BN surface are not completely encapsulated by epoxy resin, imparting nanoscale roughness to the coating surface. This is conducive to imparting hydrophobicity to the coating.

### Thermal Properties of the Composite Coatings

#### Thermal Conductivity of the Composite Coatings

**Fig. 3:**
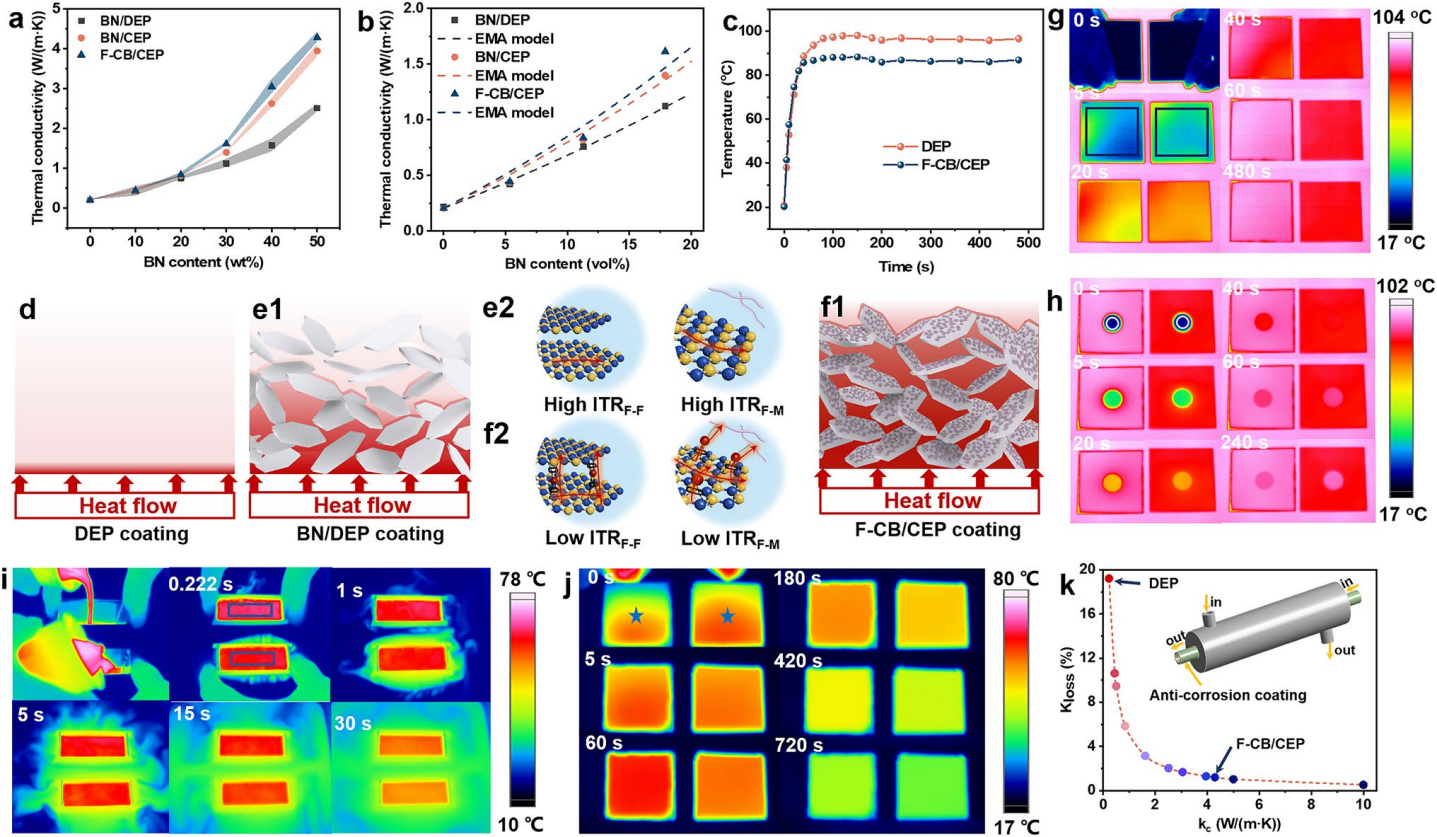
**a** Variation of TC of different coatings with BN content. **b** TC fitting results by EMA model of different coatings. **d-f** Illustration of thermal conduction mechanism of **d** DEP, **e1, e2** BN/DEP and **f1, f2** F-CB/CEP coatings. "F-F" and "F-M" represents the filler-filler and filler-matrix interface. **c** Average surface temperature in the marked areas and **g** infrared thermal images versus time of DEP coating (left) and F-CB/CEP coating (right) after placing the coated steel plates simultaneously on a heating platform preheated to 100 °C. Infrared thermal images versus time of **h** same stainless steel sheet on the above preheated samples, **i** hot water in the aluminum boxes coated with DEP coating (up) and F-CB/CEP coating (down) when cold water at room temperature was used as cooling medium, and **j** the side of the aluminum boxes coated with DEP coating (left) and F-CB/CEP coating (right) filled with hot water when air was used as cooling medium. **k** Variation in *K*_loss_ values for the coated heat exchanger at various *k*_c_

Figure 3a presents the variation of TC for composite coatings with different BN contents. Obviously, TC variations reveal the following characteristics: On one hand, using CMA as curing agent for epoxy resin does not increase the TC of pure epoxy coating, while the TC of BN/CEP is higher than that of BN/DEP at the same BN content. So it can be speculated that the synergistic effect of CMA and BN could cause the TC enhancement of BN/CEP and F-CB/CEP coatings. On the other hand, it is not until the BN content reaches 30 wt% that the TC of different coatings show conspicuous differences. And this difference becomes more apparent at higher BN content. This proves that the hybrid F-CB heat conduction network facilitated by the connection of h-CMAs nanoparticles causes more effective heat transfer than the single BN heat conduction network. This is mainly because that as filler content increases, the highly thermally conductive filler network dominates the improvement of TC [29]. For the hybrid F-CB network, h-CMAs could act as bridges between adjacent BN sheets by the virtue of "cation-π" interaction. This could reduce the ITR and provide efficient channels for phonon transport between the adjacent BN sheets compared to pure epoxy, resulting in a significant improvement in TC of F-CB/CEP coating compared with other coatings at the same BN loading [30, 31]. Specifically, in comparison with the low TC of 0.76, 0.82, and 0.84 W m^−1^ K^−1^ for BN/DEP, BN/CEP, F-CB/CEP coatings, respectively, when the BN content is 20 wt%, the TC of the above coatings reach 2.51, 3.95, and 4.29 W m^−1^ K^−1^ after introducing 50 wt% BN. According to the above analysis, the enhanced heat conduction mechanism of F-CB/CEP coating is shown and discussed in detail in Fig. 3d–f and Text S8.

#### Heat Conduction Properties of the Composite Coatings

To intuitively present the heat absorption and heat dissipation capabilities of F-CB/CEP coating compared to pure epoxy coating, infrared camera was used to monitor the surface temperature variation with time of the coated steel plates during the heating process on a heating table preheated to 100 °C. The results are shown in Fig. 3c, g. Clearly, faster temperature rise can be observed on F-CB/CEP coating compared to DEP coating during the initial heating process (before 20 s), indicating that F-CB/CEP coating has better heat absorption capability, which can quickly absorb the heat transferred from the bottom steel plate. Subsequently, the temperature of F-CB/CEP coating continues to rise until stabilizing at around 86 °C after 40 s. However, the surface temperature of DEP coating does not become stable until 80 s, and the final temperature is about 10 °C higher than that of F-CB/CEP coating. Such great contrast can be attributed to the superior heat dissipation capability of the F-CB/CEP coating, which allows the heat absorbed by the coating to dissipate quickly. This is further confirmed by the temperature variation of the stainless steel disk placed on the above samples surface over time as shown in Figs. 3h and S4a (the samples are pre-stabilized on the heated platform for 20 min). Even though the F-CB/CEP coating exhibits a lower surface temperature, the stainless-steel disk on its surface experiences a quicker temperature rise than that on the DEP coating, revealing that F-CB/CEP coating could transfer more heat to stainless steel disk on its surface compared to DEP coating. These emphasize the superior thermal response ability of F-CB/CEP coating.

#### Thermal Management Performance of the Coated Metal Substrates

For better and more intuitively evaluation of the actual thermal management performance of metallic materials covered by F-CB/CEP coating, we used double-sided coated aluminum sheets to prepare aluminum boxes (without top lid) and used them to contain hot water. The hot water was served as a transient heat source, while the temperature variation of the water or boxes was observed during the liquid cooling or gas cooling processes. First, cold water at room temperature was used as the cooling medium (optical image is shown in the inset in Fig. S4b). The aluminum boxes coated with DEP (up) and F-CB/CEP (down) coatings were placed in a container filled with cold water in advance, and then two cups of hot water with same volume and temperature were simultaneously poured into the aluminum boxes. The moment when all the hot water were poured into the aluminum boxes was recorded as 0 s and the temperature change of water in the aluminum boxes was captured from the top. Figures 3i and S4b depict that the hot water in F-CB/CEP coated aluminum box exhibits a faster cooling rate, especially during the initial observation. Then, infrared thermography was also used to observe the temperature variation on the side of coated aluminum boxes when air was used as the cooling medium (Figs. 3j, S4c and Text S10). These verify the superior thermal management capabilities of F-CB/CEP coated metallic materials.

#### Application Case Calculation of the Composite Coatings

Theoretical calculations of the overall heat transfer coefficient loss (*K*_loss_) of the coated heat exchanger compared with the original heat exchanger were performed to quantitatively analyze the effect of coating on heat transfer capacity of the coated metallic materials. The specific calculation process is shown Text S11 and the variation of *K*_loss_ values with the different TC values of coating (*k*_c_) is shown in Fig. 3k and Table S1. According to our survey, the TC of most reported polymer-based anti-corrosion coatings is lower than 0.5 W m^−1^ K^−1^. Results show that the *K*_loss_ is 9.47% at the *k*_c_ of 0.5 W m^−1^ K^−1^. And the lower the *k*_c_, the more pronounced increase in *K*_loss_. The *K*_loss_ is up to 19.21% at *k*_c_ of 0.22 W m^−1^ K^−1^ for DEP coating. Importantly, the accumulation of energy and economic losses caused by the obvious *K*_loss_ during the long-term operation cannot be ignored. Surprisingly, for the F-CB/CEP coating with TC of 4.29 W m^−1^ K^−1^, the *K*_loss_ value is reduced to 1.20%, causing efficient energy and economic cost savings.

### Corrosion Protection Properties of the Composite Coatings

#### EIS Tests of the Composite Coatings

**Fig. 4:**
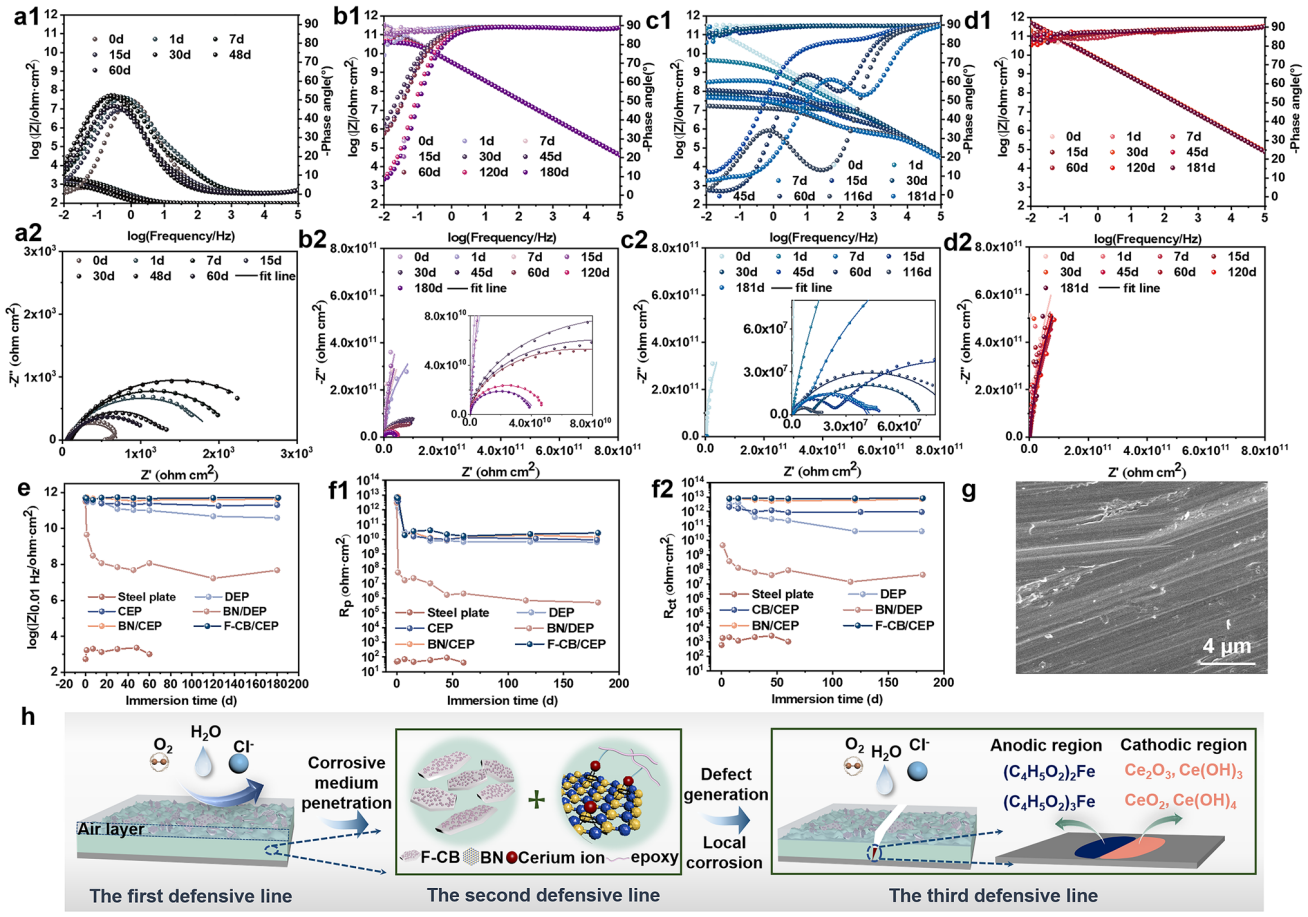
Bode plots and Nyquist plots for **a1, a2** bare steel plate, **b1, b2** DEP, **c1, c2** BN/DEP and **d1, d2** F-CB/CEP coatings, respectively. The evolution of **e** |Z|_0.01 Hz_, **f1** *R*_p_ and **f2** *R*_ct_ of bare steel plate and different coatings during the immersion in 3.5 wt% NaCl solution. **g** SEM image of the steel plate surface underneath F-CB/CEP coating after 150 days of immersion in 3.5 wt% NaCl solution. **h** Illustration of the protective mechanism of F-CB/CEP coating

Corrosion resistance properties of the composite coatings were evaluated by EIS measurements obtained by exposing the samples to 3.5 wt% NaCl solution at different immersion time. The obtained Bode plots and Nyquist plots are shown in Figs. 4a–d and S5. Generally, the higher impedance modulus at low frequency (|Z|_0.01 Hz_) and phase angle values, and the larger capacitive arc, that is, the better anti-corrosion properties of the coating [24]. Hence, the evolution of |Z|_0.01 Hz_ is also summarized in Fig. 4e. As depicted by the low |Z|_0.01 Hz_ and phase angle values, as well as the extremely small capacitive arc in Fig. 4a, e, the bare steel plate corrodes rapidly. So we did not soak it for a longer period of time.

As shown in Figs. 4b and S5a, the DEP and CEP coatings show some protection for metal substrate. Notably, the |Z|_0.01 Hz_ values of CEP coating are always higher than those of DEP coating and are maintained above 10^11^ Ω cm^2^ at the whole immersion period, while the |Z|_0.01 Hz_ of DEP coating decreases gradually with the prolongation of soaking time (Fig. 4e). This indicates that CMA as curing agent can significantly enhance the long-term corrosion resistance of epoxy coating. This can be explained by the denser crosslinking network within the coating and effective corrosion inhibition provided by CMA in the CEP coating [32]. Moreover, as depicted in Figs. 4c and S5b, the superior long-term anti-corrosion performance of BN/CEP coating than BN/DEP coating also evidences this. Besides, it is worth emphasizing that the |Z|_0.01 Hz_ and capacitance arc of BN/DEP coating show rapid decrease with increasing time. This is associated with apparent defects within the coating, which lead to rapid penetration of corrosive medium.

Surprisingly, EIS measurements of F-CB/CEP coatings with different BN contents performed after about 180-day immersion still show well resistive features, similar to those presented just after preparation (Figs. 4d and S5c–f). Notably, among these coatings, F-CB/CEP coating with 50 wt% BN exhibits optimal corrosion resistance, including consistently highest |Z|_0.01 Hz_ and phase angle values at low frequency region, as well as the maximum capacitive arc throughout the immersion time. Meanwhile, even after 181 days of immersion, there is still no breakpoint frequency (*f*_b_) in the Bode plots of the F-CB/CEP coating, and |Z|_0.01 Hz_ is maintained at 5.1 × 10^11^ Ω cm^2^. This indicates that the coating has not delaminated and has good adhesion to the substrate. Besides, as shown in Fig. 4e, the slightly rising |Z|_0.01 Hz_ values at the later stage of immersion proves the active corrosion inhibition capacity of F-CB/CEP coating, which allows for an extension of service time for the coated metallic substrate.

To further describe corrosion protection capacity and better understand the corrosion protection mechanism of F-CB/CEP coating, the obtained EIS results were fitted with equivalent circuits illustrated in Fig. S6a, b. In equivalent circuits, *R*_s_, *R*_p_, and *R*_ct_ separately imply the solution resistance, coating resistance, and charge-transfer resistance. CPE_c_ and CPE_dl_ denote the coating constant phase element and double layer constant phase element, respectively [33]. The higher *R*_p_ and *R*_ct_ values represent the better corrosion resistance of the coating. Hence, the fitted *R*_p_ and *R*_ct_ of different coatings are summarized in Figs. 4f1, f2 and S6c1, c2. All the BN/CEP coating and F-CB/CEP coatings with different BN contents exhibit high *R*_p_ and *R*_ct_ values, revealing excellent anti-corrosion performance and echoing the EIS results. Notably, among all the coatings, F-CB/CEP coating with 50 wt% BN exhibits higher *R*_p_ value than other coatings. This is related to the good physical barrier effect provided by the synergistic effect of hydrophobic surface, numerous well-dispersed 2D fillers, and dense coating structure. Besides, the *R*_ct_ value which reflects the corrosion reaction at the coating/metal interface of F-CB/CEP coating is also always higher than those of other coatings, especially at the later soaking phase. This clearly illustrates the corrosion inhibition provided by the cerium ions and methacrylate ions in the F-CB/CEP coating. Furthermore, SEM morphology of the steel plate surface protected by the F-CB/CEP coating after soaking in 3.5 wt% NaCl solution for 150 days was observed (Fig. 4g). It can be seen that there are no obvious corrosion products on the surface of the steel plate with clear grinding marks, further supporting excellent long-term anti-corrosion of the F-CB/CEP coating.

#### Corrosion Inhibition of CMA and F-CB

Corrosion inhibition of CMA and F-CB in F-CB/CEP coating was investigated by potentiodynamic polarization test (see details in Text S12). As shown in Fig. S7 and Table S2, both the SEM results of steel plates immersed in the 3.5 wt% NaCl solution without filler (blank solution), with CMA, and with F-CB and CMA for different time and the corresponding polarization curves after 47 h of immersion indicate that the F-CB and CMA could effectively reduce the corrosion rate and enhance corrosion resistance by forming the insoluble cerium oxide/hydroxide species and iron methacrylate complexes on the steel plate surface. Based on the above discussion, the F-CB/CEP coating exhibits outstanding long-term anti-corrosion performance due to the effective triple defense integrated by surface repelling, self-barrier, and double corrosion inhibition. The corrosion protection mechanism is illustrated schematically in Fig. 4h and discussed in detail in Text S13 to understand the anti-corrosion performance of F-CB/CEP coating more visually and comprehensively.

### Wettability and Self-cleaning Properties of the Composite Coatings

**Fig. 5:**
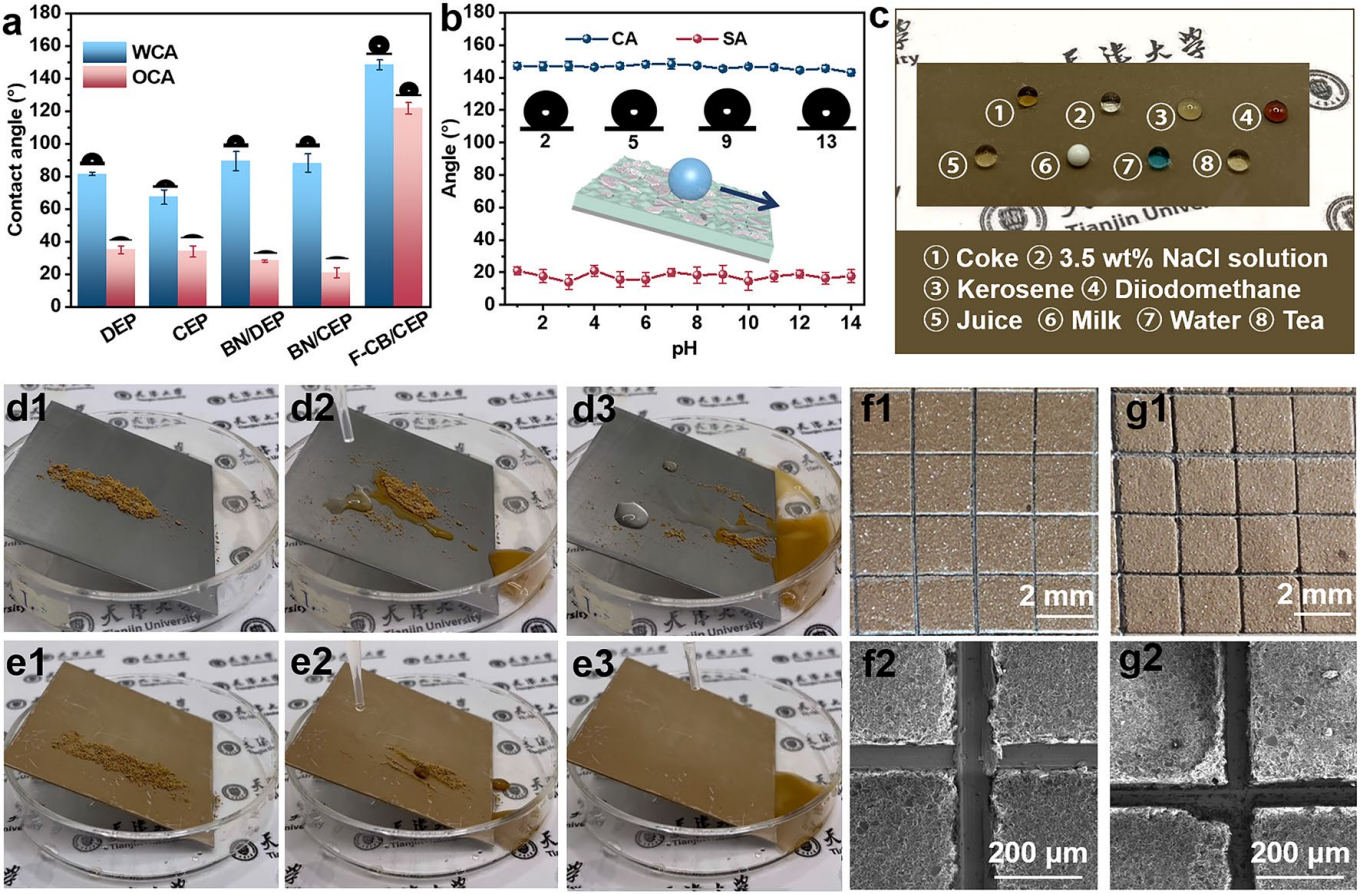
**a** WCA and OCA of different coatings. **b** Influence of solution pH on the wettability of F-CB/CEP coating. **c** Optical photographs of different droplets on F-CB/CEP coating. Optical photographs during the self-cleaning tests of **d1-d3** DEP and **e1-e3** F-CB/CEP coatings. Optical photographs and SEM images of the **f1, f2** original F-CB/CEP coating and **g1, g2** F-CB/CEP coating immersed in 3.5 wt% NaCl solution for 302 days after the adhesion test

The surface wettability of various coatings was investigated via the contact angle measurements. The results including the water contact angles (WCAs), oil contact angles (OCAs), contact angles (CAs) and sliding angles (SAs) of other droplets are presented in Fig. 5a, b. Compared to other coatings those do not exhibit any hydrophobic properties (WCAs are lower than 90°, and water droplets are completely pinned on the inclined surfaces except for pure epoxy coatings with the WSAs higher than 50°), the WCA of F-CB/CEP coating increases to 148.35° ± 3.02° and WSA of F-CB/CEP coating reduces to 20° ± 2.5°, exhibiting good hydrophobic properties. This is associated with the air film formed by the numerous highly fluorinated fillers with abounding low-surface-energy groups and micro-/nano-hierarchical structure on the coating surface [34]. Meanwhile, F-CB/CEP coating also exhibits a high OCA of 121.67° ± 3.48°. Besides, the CAs of droplets at pH values from 1 to 14 on the F-CB/CEP coating are all higher than 143°, and the SAs are all lower than 22°. And Fig. 5c illustrates that various liquids including 3.5 wt% NaCl solution could retain spherical shapes on F-CB/CEP coating surface. These demonstrate that the F-CB/CEP coating has broad repellency to a wide range of liquids.

In addition, F-CB/CEP coating shows well self-cleaning performance. As shown in Fig. 5d, due to hydrophilicity of DEP surface, dust cannot be immediately washed away by water droplets. And then the dust is just carried away by the gravity of water droplets whereas lots of water and dust are still left on coating surface. Conversely, Fig. 5e clearly shows that water droplets can easily and quickly carry the dust away, and no "leftovers" on the F-CB/CEP surface, revealing well self-cleaning property of F-CB/CEP coating. Therefore, the hydrophobic F-CB/CEP coating is suitable for preventing the metal substrate from contamination in practical industrial applications. Table S3 lists the comparative data of the F-CB/CEP coating in our work compared to other thermally conductive anti-corrosion coatings previously reported in terms of heat conduction, anti-corrosion and wettability. Obviously, F-CB/CEP coating fully combined the advantages of F-CB, CMA, and epoxy resin shows optimal comprehensive performance, including the highest TC, |Z|_0.01 Hz_, OCA, high WCA, and the lowest *K*_loss_.

### Temperature Resistance and Adhesion of the Composite Coatings

The F-CB/CEP coating also possesses well temperature resistance and adhesion (Text S14 and S15). As shown in Fig. S9a-f, after placing in 180 °C at air environment or soaking in deionized water or solution at pH = 5 or 9 in reactor at 140 °C for 10 heating circles, or placing in constant temperature (50 °C) and humidity (95%) environment for 200 h, the F-CB/CEP coating still maintains its original shape without damage including layering, wrinkling, bubbling or cracking. Tape-peel test reveals that the F-CB/CEP coating shows a grade 1 adhesion (Fig. 5f). Moreover, Fig. 5g shows that even after long-term immersion in 3.5 wt% NaCl solution, the coating still maintains grade 1 adhesion. Accordingly, we successfully prepared a multifunctional epoxy composite coating, which has strong application prospects under diverse application conditions.

## Conclusions

In summary, an anti-corrosive F-CB/CEP coating with high TC, well corrosion inhibition, as well as water repellency for protecting the metals used as thermal management materials was fabricated by synthesizing CMA as both the epoxy resin curing agent and BN modifier, and further fluorinating modification of CB. Finally, by integrating CMA, F-CB into epoxy resin, the prepared F-CB/CEP composite epoxy simultaneously delivered a high TC (4.29 W m^−1^ K^−1^), a high |Z|_0.01 Hz_ value after 181 days of immersion (5.1 × 10^11^ Ω cm^2^), a high WCA (148.35° ± 3.02°), and a low WSA (20° ± 2.5°). In addition, infrared thermography results showed that F-CB/CEP coating could effectively maintain excellent heat transfer and heat dissipation capabilities of the protected metal substrate compared to ordinary epoxy coating. This was also confirmed by case calculation about applying the coatings to the surface of metallic heat exchange tube. Besides, good hydrophobicity made the F-CB/CEP coating exhibit additional self-cleaning capability. Therefore, our work provides, first of all, an effective and intelligent curing agent for epoxy anti-corrosion coatings. Secondly, the F-CB/CEP coating can extend service life of metallic materials without sacrificing their inherent thermal conduction performance. In addition, the well hydrophobic properties of the coating could prevent the metal substrate from contamination in practical industrial applications. And the coating possesses well adhesion and temperature resistance. The unique, scalable and multifunctional coating has strong application prospects in the development of multifunctional corrosion resistance coatings in the coming future.

### Supplementary Information

Below is the link to the electronic supplementary material.

Supplementary file 1 (DOCX 4998 KB)
