# Supplementary material for: Cerium Methacrylate Assisted Preparation of Highly Thermally Conductive and Anticorrosive Multifunctional Coatings for Heat Conduction Metals Protection
Source: Nanomicro Lett. 2023 Aug 18;15:201. doi: 10.1007/s40820-023-01163-w (PMC10439099; doi:10.1007/s40820-023-01163-w)
Supplement: Supplementary file 1 — (DOCX 4998 KB) [file 40820_2023_1163_MOESM1_ESM.docx]

Supporting Information for

**Cerium Methacrylate Assisted Preparation of Highly Thermally Conductive and Anticorrosive Multifunctional Coatings for Heat Conduction Metals Protection**

Fei Xu^1^, Peng Ye^1^, Jianwen Peng^1^, Haolei Geng^1^, Yexiang Cui^1^, Di Bao^1,2^, Renjie Lu^1^, Hongyu Zhu^3^, Yanji Zhu^3^, Huaiyuan Wang^1,4,5,*^

^1^ School of Chemical Engineering and Technology, Tianjin University, Tianjin 300350, PR China

^2^ Haihe Laboratory of Sustainable Chemical Transformations, Tianjin 300192, PR China

^3^ Tianjin Key Lab Composite & Functional Materials, School of Materials Science and Engineering, Tianjin University, Tianjin 300072, PR China

^4^ State Key Laboratory of Chemical Engineering and Collaborative Innovation Center of Chemical Science and Engineering (Tianjin), Tianjin University, Tianjin 300072, PR China

^5^ Tianjin Key Laboratory of Chemical process safety and equipment technology, Tianjin University, Tianjin 300072, PR China

*Corresponding author. E-mail: [huaiyuanwang@tju.edu.cn](mailto:huaiyuanwang@tju.edu.cn)

**Text S1 Materials**

Boron nitride (BN, lateral size is approximately 25 μm) was acquired from Dandong Rijin Technology Co., Ltd. (Dandong, China). Cerium (III) nitrate hexahydrate (Ce(NO_3_)_3_·6H_2_O) was supplied by Aladdin Biochemical Technology Co., Ltd. (Shanghai, China). Ammonium bicarbonate (NH_4_HCO_3_) was purchased from FUCHEN (Tianjin) Chemical Reagents Co., Ltd. (Tianjin, China). Methacrylic acid (MAA) was purchased from Shanghai Macklin Biochemical Co., Ltd. (Shanghai, China). 1H, 1H, 2H, 2H-perfluorodecyltriethoxysilane (FAS) was from Nanjing Quanxi Chemical Co., Ltd. (Nanjing, China). Ethyl acetate was provided by Tianjin Yuanli Chemical Co., Ltd. (Tianjin, China). D-230 (polyether amine) bought from Chuzhou Huisheng Electronic Materials Co., Ltd. (Chuzhou, China) was used as the comparative commercial curing agent for epoxy resin. Epoxy resin (E-51) was purchased from Jining Huakai Resin Co., Ltd. (Jining, China). All the reagents were used as received. Q235 carbon steels (70 mm × 70 mm × 1 mm) were used as metal substrates.

**Text S2 Characterization**

**Text S2.1 The characterization of fillers and coatings**

The morphology and element composition of fillers and coating samples were obtained by transmission electron microscope (TEM, JEM-F200) and scanning electron microscopy (SEM, Regulus 8100 or Apreo S LoVac) equipped with energy dispersive spectroscopy (EDS). The change of cerium methacrylate (CMA) crystals in epoxy matrix during the curing process was captured using a polarization microscope (Nikon H600L). And thermal performance of the coatings was tested by differential scanning calorimetry (DSC, TA Instruments, Q2000, nitrogen atmosphere). The chemical composition, crystalline structure and relative information regarding the CMA and fillers, as well as the uncured and cured coatings were detected by Fourier transformation infrared spectrometer (FT-IR spectrometer, Perkin Elmer), X-ray diffraction (XRD, D8 Advanced, Bruker) using Cu Kα radiation (λ = 1.5406 Å, 40 kV and 40 mA), ultraviolet-visible spectroscopy (UV-vis, TU-1901), X-ray photoelectron spectroscopy (XPS, Thermo Scientific K-Alpha), respectively. The roughness (Ra) of the coatings surface was tested by portable roughness tester (Mitutoyo, SJ-210).

**Text S2.2 Thermal properties measurement of the coatings**

Due to the force majeure factors (e.g., interfacial thermal resistance (ITR) between the coating and substrate interface), it is difficult to directly and accurately test thermal conductivity (TC) of the coating on substrate surface. Conversely, the TC of lumped composites is more readily available. As a result, bulk composites (thickness greater than 2.3 mm and lateral size approximately 20 mm) with same compositions as the coatings were prepared for the TC measurement. Infrared thermal imager (Ti480 Pro, Fluke) was used to capture the thermal management capabilities of the composite coatings.

**Text S2.3 Corrosion resistance properties measurement of the coatings and fillers**

To evaluate corrosion resistance properties of the coatings and fillers, electrochemical measurements, including electrochemical impedance spectroscopy (EIS) test and potentiodynamic polarization test, were conducted in 3.5 wt% NaCl solution with the electrochemical workstation (Metrohm Autolab PGSTAT302N). Conventional three-electrode system was used for tests with saturated calomel electrode as reference electrode, platinum electrode as counter electrode, and coated metal substrate with exposed area of 7.065 cm^2^ as working electrode, respectively. For EIS measurement, the electrochemical data were captured in the frequency range of 10 mHz-100 kHz with the sinusoidal perturbation of 20 mV amplitude. Before each test, samples were immersed in NaCl solution for ~25 min to stabilize the open circuit potential. For each sample, the reproducibility of the obtained test result was checked by three replicates and middle value was selected as the final reported data. The EIS data were fitted by ZSimpWin software. The potentiodynamic polarization curves of steel plates after different immersion time in 3.5 wt% NaCl solution, and 3.5 wt% NaCl solution with 50 mg/L of CMA or 1 g/L of F-CB and 50 mg/L of CMA were tested to explore the corrosion inhibition behavior of cerium ions and methacrylate ions in the coatings.

**Text S2.4 Wettability test of the coatings**

Water contact angles (WCAs) with 10 μL of water droplet, water sliding angles (WSAs) with 30 μL of water droplet, and oil contact angles (OCAs) with 10 μL of sunflower seed oil were measured on contact angle measuring system (Attention Theta Lite). And average value was recorded as the final result by testing at least four different positions of each sample. The self-cleaning tests of different coatings were performed as follows: 0.1 g of dust powders were first dispersed on the sloped coating surfaces. Then, water droplets were slowly dropped on coatings surface and the optical photographs during this process were recorded.


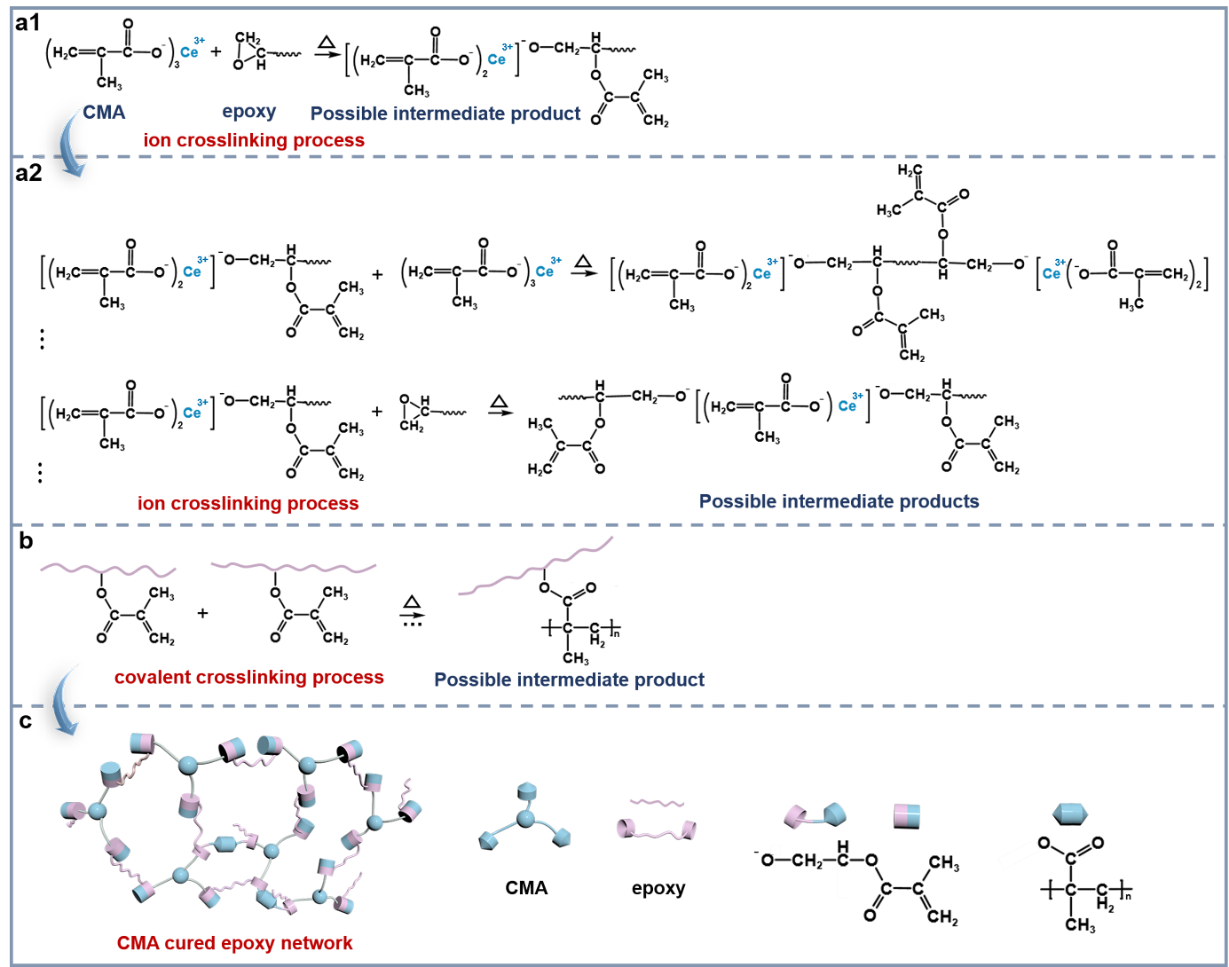


**Fig. S1** Scheme diagram for the curing process of epoxy resin cured with CMA. The **a** ion crosslinking process, **b** covalent crosslinking process, and **c** cured epoxy network

**Text S3 FT-IR characterization of MAA, Ce_2_(CO_3_)_3_ and CMA**

FT-IR spectra were tested to confirm the successful synthesis of CMA. As shown in Fig. 1f, compared with the spectrum for MAA, the absorption peaks at 1533 cm^−1^ and 1415 cm^−1^ on spectrum of CMA are scribed to the vibrations of carboxylate [S1]. Moreover, the apparently weakened absorption peak for -OH at 2980 cm^-1^, and the appearance of Ce-O absorption peak at 410 cm^-1^ on the spectrum of CMA account for the coordination of carboxylate in MAA with Ce^3+^ ions [S2]. Besides, the characteristic stretching vibrational absorption peak of C=C bond shifts from 1636 cm^-1^ for MAA to 1645 cm^-1^ for CMA, indicating that the C=C bond was not open to participate in the coordination of cerium, but was shifted due to the effect of receiving the cerium coordination. These indicate the successful synthesis of CMA.

**Text S4 The feasibility of CMA as curing agent for epoxy resin**

FT-IR measurements evince that compared with epoxy and uncured CEP, FT-IR spectrum for cured CEP exhibits a significantly weaker characteristic peak at 914 cm^-1^ for epoxy group. Meanwhile, the appearance of new peak at 1738 cm^-1^ [corresponding to the ester](https://cn.bing.com/dict/search?q=ester&FORM=BDVSP6&cc=cn) [group](https://cn.bing.com/dict/search?q=group&FORM=BDVSP6&cc=cn) stretching vibration confirms the presence of ester group in the cured epoxy network (Fig. 1g). These indicate that the ring-opening reaction between CMA and epoxy resin was occurred at high temperature, resulting in the successful curing of epoxy resin. The specific might reactions during the curing of epoxy resin with CMA as curing agent are shown in Fig. S1. It is worth emphasizing that some of the adjacent C=C bonds on methacrylate might undergo polymerization at high temperatures to form covalent crosslinking network, but usually in very small yields [S3]. Therefore, we will not discuss this too much here.

**Text S5 XRD characterization of the fillers**

XRD pattern of CMA shows diffraction double peaks at 2θ = 6.23° and 7.82°, corresponding to the typical characteristic peak positions of carboxylates (Fig. 2d). For XRD pattern of CB, the relative peak intensity of the diffraction double peaks has changed compared with that of the original synthetic CMA, which may be related to the morphological change from irregular agglomerates of original CMA to homogeneous nanoparticles on BN surface [S4, S5]. In addition, it is noteworthy that the XRD characteristic peaks of F-CB are almost unchanged compared with those of CB, proving that fluorination modification would not affect crystal structures of CB.

**Text S6 XPS characterization of the fillers**

For the Ce3d, based on notation proposed by Burroughs et al, the bands labeled "v" and "u" correspond to the 3d_5/2_ ionization and 3d_3/2_ ionization, respectively. Ce^4+^ ions are associated with peaks labeled u, v, u", v", u"', v"', while Ce^3+^ ions are associated with peaks labeled u_0_, v_0_, u', v' [S6, S7]. As depicted in Fig. 2f3, it is clear that the high-resolution spectra of Ce3d for CMA only show the peaks (u_0_, v_0_, u', v') associated with Ce^3+^ ions, indicating that all the cerium ions in the synthesized CMA exist in the trivalent state. Remarkably, high-resolution spectra of Ce3d for CB show characteristic peaks associated with both the Ce^3+^ and Ce^4+^ ions. This indicates that part of Ce^3+^ ions was oxidized to Ce^4+^ ions during the modification of BN. And the proportion of Ce^4+^ ions is calculated by obtaining the ratio of the summation of u, v, u'', v'', u''', and v''' areas to the total areas, which yields a value of 55.08%. Therefore, after modification process, nanoparticles on BN surface are not pure CMA, but exist in the form of hybrids. For the convenience of expression, these hybrids are named as h-CMAs in our work. Moreover, compared with CMA, the peaks assigning to u_0_, v_0_, u' and v' for CB all shift to lower binding energies. Researches have shown that the chemical environment of atoms participating in particular interactions would be changed [S8, S9]. Therefore, this further proves the existence of "cation-π" interaction between BN and cerium ions. For the XPS results of F-CB, the appearance of F1s and Si-O characteristic peaks further prove the successful modification of CB by FAS (Fig. 2f1, f2). Similarly, cerium ions in F-CB exist in the trivalent and tetravalent coexisting state, and calculation result reveals little change in Ce^4+^ ions content (54.84%) compared with that of CB, indicating that fluorination treatment would not affect the valence state of cerium ions.

**Text S7 SEM images of the composite coatings**

Fig. S2 shows the surface and cross-sectional SEM images of F-CB/CEP coatings with different contents of BN (10 wt%-50 wt%). Fig. S2a1-e2 show that as filler content increases, the surface roughness of F-CB/CEP coating gradually increases, which is also confirmed by the increased surface roughness (Ra) value in Fig. S3a. This is conducive to imparting hydrophobicity to the coating surface. For the cross-sectional images of different composite coatings with the same 50 wt% BN content, Fig. S3b1 and b2 clearly shows that there are obvious interfacial defects between BN and epoxy matrix in the BN/DEP coating. For the BN/CEP and F-CB/CEP coatings with CMA as curing agent (Fig. S3c and d), good interfacial connection between BN and epoxy matrix can be observed. And compared with BN/CEP coating, F-CB/CEP coating shows better adhesion between filler and epoxy matrix, demonstrating the effective bridging effect provided by h-CMAs nanoparticles in the coating.


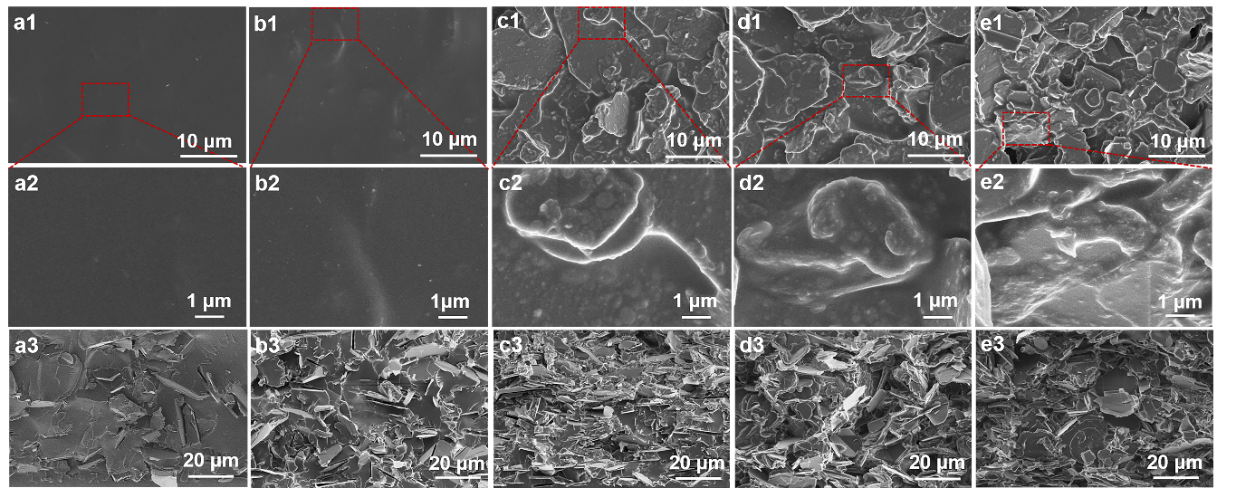


**Fig. S2** Surface and cross-sectional SEM images of F-CB/CEP coatings with **a1-a3** 10 wt%, **b1-b3** 20 wt%, **c1-c3** 30 wt%, **d1-d3** 40 wt% and **e1-e3** 50 wt% BN contents, respectively


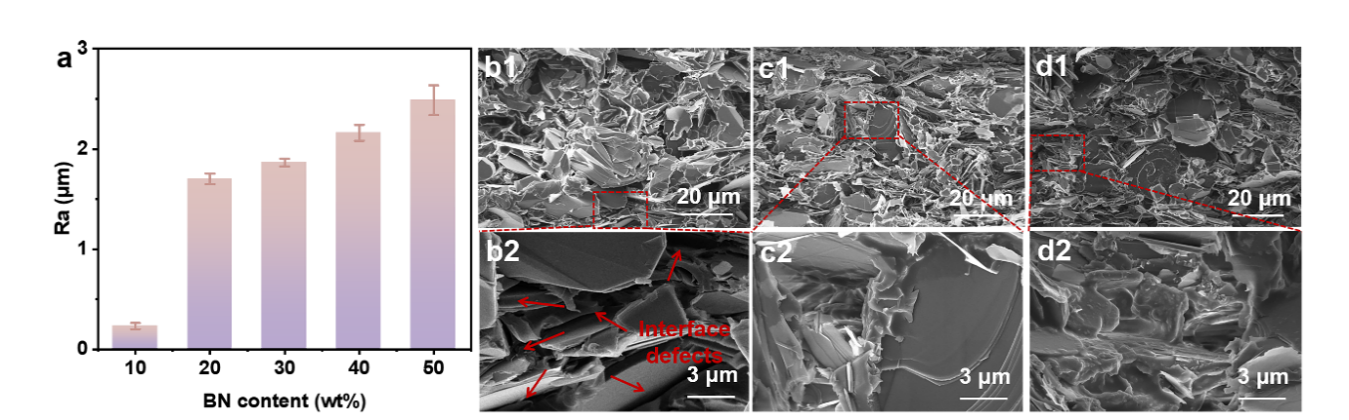


**Fig. S3** **a** Ra value of F-CB/CEP coatings with BN contents from 10 wt% to 50 wt%. Cross-sectional SEM images of **b1, b2** BN/DEP, **c1, c2** BN/CEP, **d1, d2** F-CB/CEP coatings with the same 50 wt% BN content

**Text S8 The** **enhanced heat conduction mechanism of F-CB/CEP coating**

Such a high TC of 4.29 W/(m·K) for the F-CB/CEP coating, which is nearly 19 times higher than that of DEP coating (0.22 W/(m·K)), is firstly attributed to the compact stacking of F-CB hybrids at 50 wt% BN content compared with the DEP coating in Fig. 3d (as shown in Fig. 3f1). This allows most of the heat to be transferred quickly along the highly thermally conductive F-CB pathways. For BN/DEP coating (Fig. 3e1 and e2), even though the BN content is high enough to form interconnected BN pathways, there still exists a small amount of epoxy matrix between adjacent BN, which cannot guarantee the formation of dense, high-quality thermally conductive pathways. Conversely, cerium ions could act as bridges between adjacent BN sheets by the virtue of "cation-π" interaction for F-CB/CEP coating. This could reduce the ITR and provide efficient channels for phonon transport between the adjacent BN sheets compared to pure epoxy, resulting in more rapid heat transfer between BN sheets (Fig. 3f2). Besides, as mentioned above, the h-CMAs nanoparticles also tightly bridge the BN and epoxy matrix (Fig. 3f2), causing the rapid heat transfer between BN and epoxy matrix as well. This is confirmed by the significantly reduced ITR value between BN and epoxy matrix (*R*_b_) of F-CB/CEP coating (3.65 × 10^-8^ m^2^·K·W^-1^) compared with that of BN/CEP coating (3.15 × 10^-7^ m^2^·K·W^-1^) and BN/DEP coating (1.16 × 10^-6^ m^2^·K·W^-1^) fitted by effective media approximation (EMA) model (Fig. 3b, see more details in Text S9). The results also show that using CMA as curing agent is more effective in reducing the ITR between BN and epoxy matrix compared to the common commercial D230 curing agent. This is because that methacrylate end of CMA would be involved in the curing of epoxy resin, and there exists "cation-π" interaction between BN and cerium ions in the epoxy matrix, which would lead to a relatively good interfacial connection between BN and epoxy matrix compared with the BN/DEP coating. The cross-sectional SEM images of the coatings in Fig. S3b, c could prove this.

**Text S9 The EMA model fitting process**

The EMA model was used to quantificationally analyze the interfacial strengthening effect between epoxy matrix and BN [S10]. According to EMA model, the following equation is used to get the TC value of the composites:

$\text{K}^{\text{*}}\text{=}\text{K}_{\text{m}}\frac{\text{3+f}\text{[}\text{2β}_{\text{11}}\left( \text{1-}\text{L}_{\text{11}} \right)\text{+}\text{β}_{\text{33}}\left( \text{1-}\text{L}_{\text{33}} \right)\text{]}}{\text{3-f}\text{[}\text{2β}_{\text{11}}\text{L}_{\text{11}}\text{+}\text{β}_{\text{33}}\text{L}_{\text{33}}\text{]}}$ (1)

With

$\text{β}_{\text{ii}}\text{=}\frac{\text{K}_{\text{ii}}^{\text{c}}\text{-}\text{K}_{\text{m}}}{\text{K}_{\text{m}}\text{+}\text{L}_{\text{ii}}\text{(}\text{K}_{\text{ii}}^{\text{c}}\text{-}\text{K}_{\text{m}}\text{)}}$ (2)

$\text{K}_{\text{ii}}^{\text{c}}\text{=}\frac{\text{K}_{\text{p}}}{\text{1+γ}\text{L}_{\text{ii}}\text{K}_{\text{p}}\text{/}\text{K}_{\text{m}}}$ (3)

$\text{γ=}\left( \text{1+2p} \right)\text{R}_{\text{b}}\text{K}_{\text{m}}\text{/}\text{a}_{\text{3}}\text{, for }\text{p}\text{≤1}$ (4)

$\text{L}_{\text{11}}\text{=}\text{L}_{\text{22}}\text{=}\frac{\text{p}^{\text{2}}}{\text{2(}\text{p}^{\text{2}}\text{-1)}}\text{+}\frac{\text{p}}{\text{2}\left( \text{1-}\text{p}^{\text{2}} \right)^{\text{3}/\text{2}}}\text{cos}^{\text{-1}}\text{p, for p}\text{＜}\text{1}$ (5)

$\text{L}_{\text{33}}\text{=1-2}\text{L}_{\text{11}}$ (6)

$\text{p=}\text{a}_{\text{3}}\text{/}\text{a}_{\text{1}}$ (7)

where *K^*^*, *K*_m_ and *K*_p_ represent the TC of composite, matrix, and filler, respectively. *f* stands for fillers' volume fraction. *L*_ii_ is geometrical factor which depends on the filler shape, in which 11 and 33 respectively represent the in-plane and through-plane direction. And *a_1_* and *a_3_* represent the lateral and longitudinal size of the filler, respectively. For the BN platelet in this work, *a_1_* is approximately 25 μm and *a_3_* is around 0.75 μm. In this case, the *R*_b_, is the sole unknown parameter in the above computation of $\text{K}^{\text{*}}$. By fitting the experimental TC data to the predicted outcomes, the *R*_b_ value can be obtained.


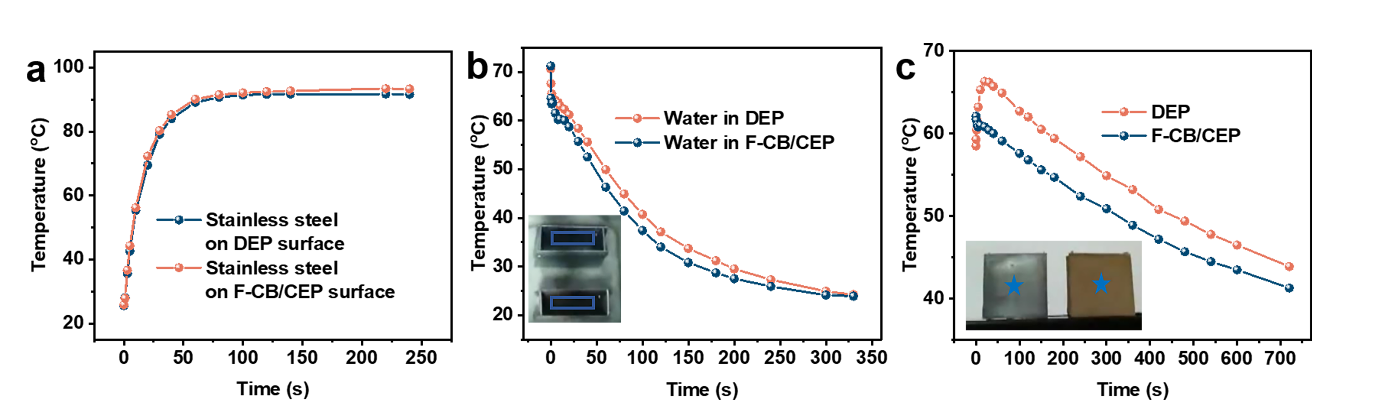


**Fig. S4 a, b** The average surface temperature variation in the marked areas in Fig. 3h and i, respectively. **c** The temperature variation at the marked sites in Fig. 3j

**Text S10 Temperature variation on the side of coated aluminum boxes when air was used as cooling medium**

Infrared thermography was used to observe the temperature variation on the side of coated aluminum boxes when air was used as the cooling medium (Fig. 3j, Fig. S4c). The specific temperature change at the star marked points is shown in Fig. S4c. The temperature on the side of the F-CB/CEP coated aluminum box first slightly increases in just 1 s with time and then decreases rapidly, exhibiting a fast temperature response. In clear contrast, hot water causes the temperature of DEP coated aluminum box to increase significantly and to decrease slowly with time. These reveal the faster heat absorption and heat dissipation capabilities of the F-CB/CEP coating, corresponding to faster thermal responsiveness.

**Text S11 The case calculation of the overall heat transfer coefficient loss (*K*_loss_) of the coated heat exchanger with different TC of the coated coating (*k*_c_)**

Appling anti-corrosion coatings on metal heat exchange tube surfaces is a typical application which needs to protect the metal device from corrosion while maintaining its original heat transfer capability. Therefore, typical shell-and-tube metal heat exchanger was used as an example to demonstrate the potential of F-CB/CEP coating in maintaining the heat transfer capability of the coated metallic materials. Based on the pertinent contents of Chapter 6 (Heat exchangers) in the *Chemical fluid flow and heat transf*er [S11], the heat transfer coefficient (*K*) and *K*_loss_ values of the heat exchanger were calculated to quantify the effect of *k*_c_ on the heat transfer capacities of the coated metal heat exchanger by assuming that the anti-corrosion coating is applied to the outer surface of the heat exchange tube. The details about the calculation are shown below.

The heat exchange tube is made of a steel tube with an outer diameter (*d*_o_) of 25 mm and an inner diameter (*d*_i_) of 20 mm. Hence, the wall thickness (*b*) is 2.5 mm. And the TC of the heat exchange tube wall (*k*_t_) is 45 W/(m·K). The tube is filled with cold fluid (water), and its convection heat transfer coefficient (*h*_i_) is 3490 W/(m^2^·K). Meanwhile, the heat fluid (oil) flows outside the tube with convection heat transfer coefficient (*h*_o_) of 258 W/(m^2^·K). Moreover, both sides of the heat exchange tube would become fouled after extended usage, causing additional thermal resistance. The thermal resistance for fouling on the inside (*R*_si_) and outside (*R*_so_) are 0.00025 m^2^·K/W and 0.000172 m^2^·K/W, respectively. In practical industrial applications, the thickness of the anti-corrosion coating is usually above 200 μm. Therefore, here, it is assumed that the thickness of coating (*d*_c_) is 250 μm, and the heat transfer process is a steady state process. To simplify the calculations, we assume that applying the coating does not affect the convection heat transfer coefficient and the thermal resistance of fouling. Hence, based on the principle of superposition of series thermal resistance, the *K* value can be computed through the follow formula:

$\text{K=}\frac{\text{1}}{\frac{\text{d}_{\text{o}}}{\text{h}_{\text{i}}\text{d}_{\text{i}}}\text{+}\text{R}_{\text{si}}\frac{\text{d}_{\text{o}}}{\text{d}_{\text{i}}}\text{+}\frac{\text{bd}_{\text{o}}}{\text{k}_{\text{t}}\text{d}_{\text{m}}}\text{+}\text{R}_{\text{so}}\text{+}\frac{\text{1}}{\text{h}_{\text{o}}}\text{+}\frac{\text{d}_{\text{c}}}{\text{k}_{\text{c}}}}$ (8)

Where *d*_m_ is the average diameter of the tube. The *K* value when no coating is applied on the outside surface of the heat exchange tube (*K*_0_) is obtained to be 209.2 W/(m^2^·K). And the *K*_loss_ of the coated heat exchanger can be calculated as:

$\text{K}_{\text{loss}}\text{=}\frac{\text{K}_{\text{0}}\text{-}\text{K}_{\text{c}}}{\text{K}_{\text{0}}}\text{×100\%}$ (9)

Where *K*_c_ is the *K* value when the anti-corrosion coating is coated on the outside surface of the heat exchange tube. The calculated *K*_c_ and *K*_loss_ values compared with the original uncoated heat exchanger with the variation of *k*_c_ are summarized in Table S1 and Fig. 3k.

**Table S1** Variation of the calculated *K_c_* and *K*_loss_ values with the *k*_c_

| *k*_c_ (W/(m·K)) | *K*_c_ (W/(m^2^·K)) | *K*_loss_ (%) |
| --- | --- | --- |
| 0.22 | 169.02 | 19.21 |
| 0.44 | 186.98 | 10.62 |
| 0.50 | 189.39 | 9.47 |
| 0.84 | 196.94 | 5.86 |
| 1.61 | 202.62 | 3.14 |
| 2.51 | 204.93 | 2.04 |
| 3.05 | 205.68 | 1.68 |
| 3.95 | 206.47 | 1.30 |
| 4.29 | 206.69 | 1.20 |
| 5 | 207.04 | 1.03 |
| 10 | 208.12 | 0.52 |


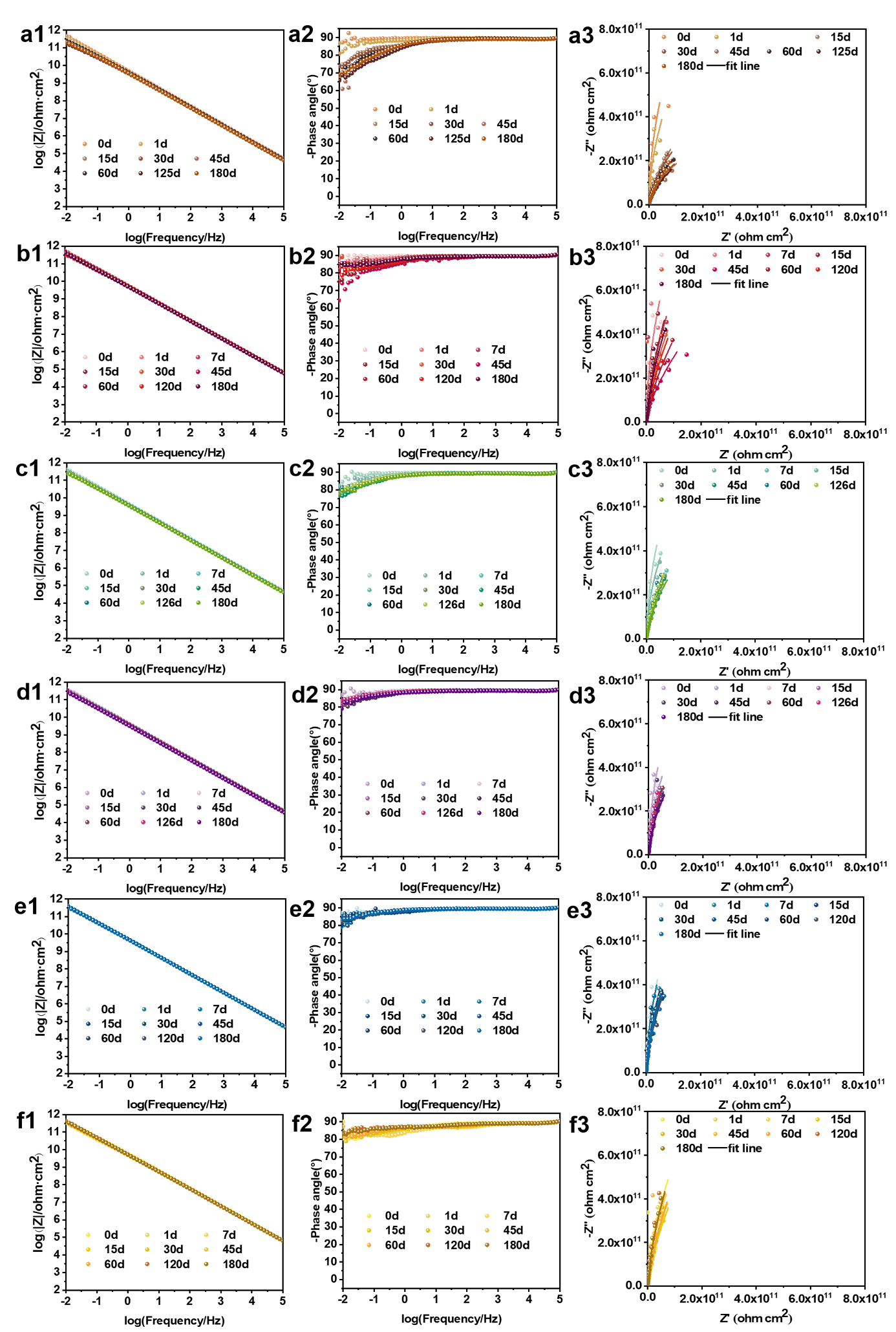


**Fig. S5** Bode plots and Nyquist plots for **a1-a3** CEP, **b1-b3** BN/CEP coatings, and F-CB/CEP coatings with different BN contents (**c1-c3** 10 wt%, **d1-d3** 20 wt%, **e1-e3** 30 wt% and **f1-f3** 40 wt%), respectively


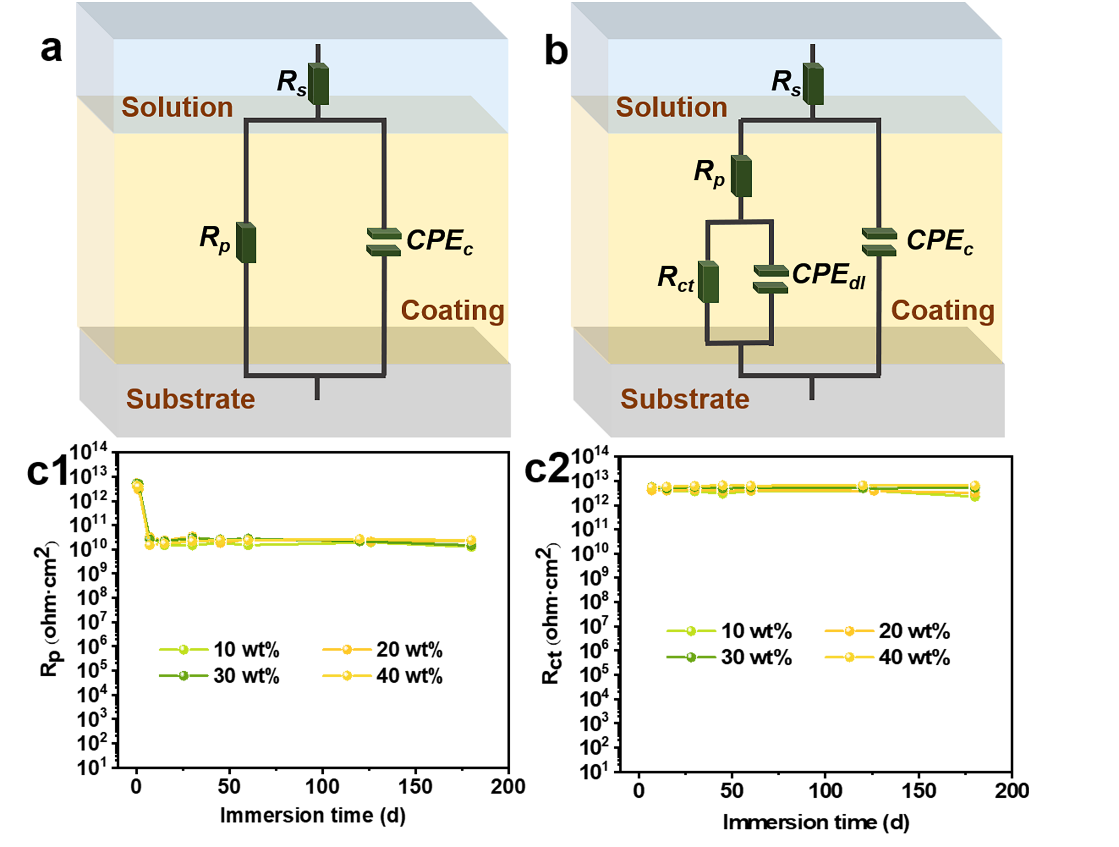


**Fig. S6 a, b** Schematic diagram of the equivalent circuits used to fit the EIS data. The evolution of **c1** R_p_ and **c2** R_ct_ of F-CB/CEP coatings with different BN loadings during the immersion in 3.5 wt% NaCl solution

**Text S12 The corrosion inhibition of F-CB and CMA in the F-CB/CEP coating**

To further investigate and prove the corrosion inhibition of F-CB and CMA in F-CB/CEP coating, bare steel plate was immersed in 3.5 wt% NaCl solution without filler, with CMA, and with F-CB and CMA for different time to record the SEM images of corresponding corrosion products and the potentiodynamic polarization curves. The results are shown in Fig. S7. Fig. S7a1-a3 show that corrosion products on the steel plate dipped in blank 3.5 wt% NaCl solution gradually increase with immersion time and form large clusters after soaking for 47 h. For steel plate immersed in salt solution containing CMA (Fig. S7b1-b3), after immersion for 23 h, the appearance of Ce element (2.11 wt%) and the increase of O and C elements prove the formation of insoluble cerium oxide/hydroxide species and iron methacrylate complexes on the steel plate surface. And when the immersion time reaches 47 h, denser protective layer is formed on the steel plate surface. Similar changes are also observed for steel plate soaked in salt solution containing F-CB and CMA. It is worth emphasizing that after immersing for 23 h, the EDS result of immersed steel plate surface show the appearance of F element, while the contents of Ce and O elements further increase. Polarization curves after 47 h of immersion shown in Fig. S7a4-c4 demonstrate that the F-CB and CMA affect both the anodic and cathodic branches of polarization curve. The extracted relevant data are represented in Table S2. Obviously, compared with the blank solution, with the addition of F-CB and CMA, the corresponding polarization curve moves to lower corrosion current density (*i*_corr_) and higher corrosion potential (*E*_corr_), representing a lower corrosion rate and better corrosion resistance [S12, S13].


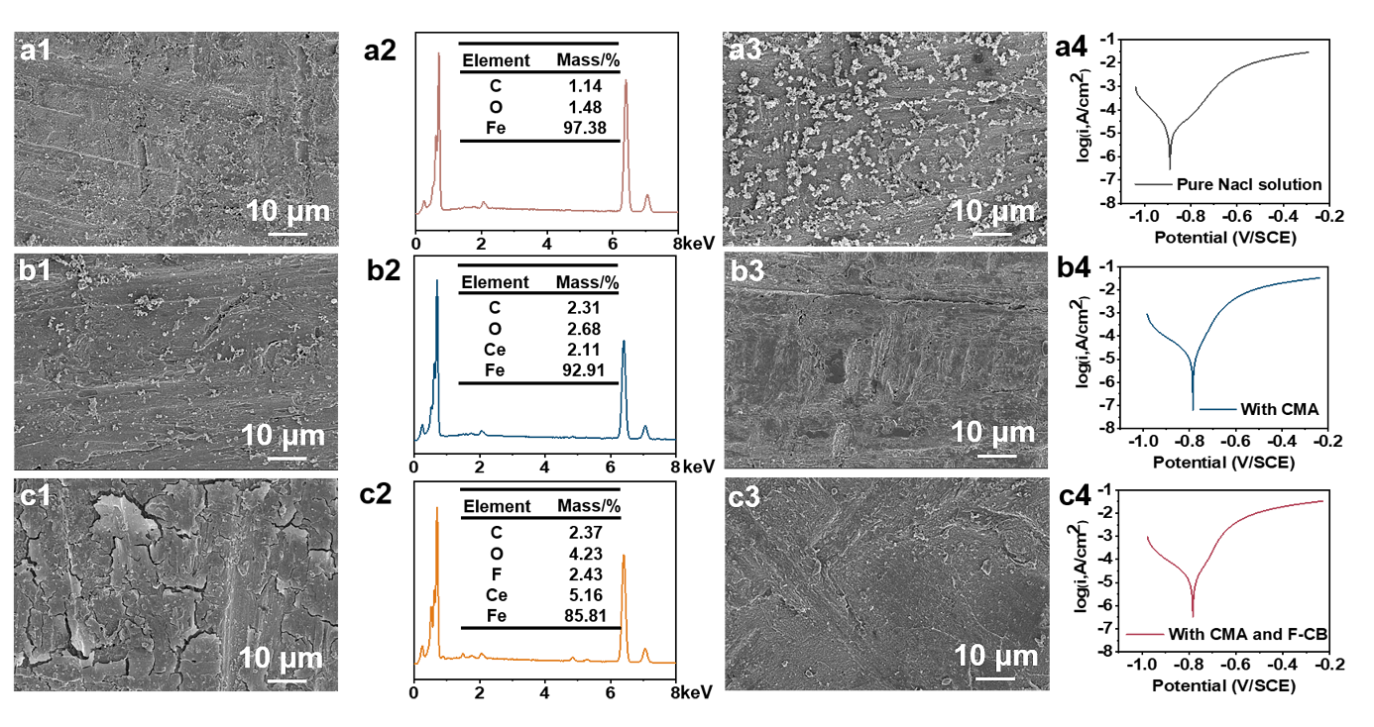


**Fig. S7** SEM images and EDS analysis of bare steel plates immersed in the 3.5 wt% NaCl solution **a** without filler (blank solution), **b** with CMA, **c** with F-CB and CMA after **a1, a2, b1, b2, c1, c2** 23 h and **a3, b3, c3** 47 h of immersion. **a4, b4, c4** Polarization curves of the above steel plates after 47 h of immersion

**Table S2** The Tafel extrapolated relevant corrosive parameters

| Sample | *E*_corr_  (V/SCE) | *i*_corr_  (A/cm^2^) | Corrosion rate  (mm/year) | η (%) |
| --- | --- | --- | --- | --- |
| Blank NaCl solution | -0.889 | 5.879E×10^-5^ | 0.097 | / |
| With CMA | -0.785 | 1.349E×10^-5^ | 0.022 | 77.06 |
| With CMA and F-CB | -0.783 | 1.690E×10^-5^ | 0.028 | 71.25 |

η: corrosion inhibition efficiency.

**Text S13 Corrosion protection mechanism of the F-CB/CEP coating**

Corrosion protection mechanism is proposed and illustrated schematically in Fig. 4h to understand the anti-corrosion performance of F-CB/CEP coating more visually and comprehensively. The corrosion protection process mainly includes three stages: (1) In the initial immersion, the air cushion formed between electrolyte solution and coating surface (this will be described in detail in section 3.6) acts as the first physical barrier, which effectively prevents the directly contact and penetration of corrosion medium. (2) As immersion time increases, the air film on coating surface would be destroyed. In this case, coating itself served as the second defensive line comes into play when corrosive medium begins to penetrate into coating interior. On one hand, the numerous 2D F-CB exhibit obvious "labyrinth effect", effectively extending penetration pathways of the corrosive medium. On the other hand, benefitting from the well bridging effect provided by h-CMAs on BN surface, the fillers are well compatible with epoxy matrix. Further in combination with the dense crosslinking network of epoxy matrix cured by CMA, the coating exhibits well impermeability against corrosive medium. (3) With the further extension of service time, corrosive medium would penetrate through the coating and finally reach substrate surface, activating the corrosion process. In this case, cerium ions and methacrylate ions in the composite coating slowly release and diffuse to the defect sites, which would form stable passivation layer served as the third defensive line at both the cathodic and anodic sites on substrate surface (Fig. S8). This would increase the corrosion potential and "heal" defects at localized corrosion sites, preventing further corrosion reaction progress and delamination of the coating [S14-S17]. In addition, good adhesion between the coating and substrate is also conducive to the maintenance of anti-corrosion performance. This effectively prevents delamination of the coating after prolonged immersion (this will be covered in detail in Section 3.7).

**_
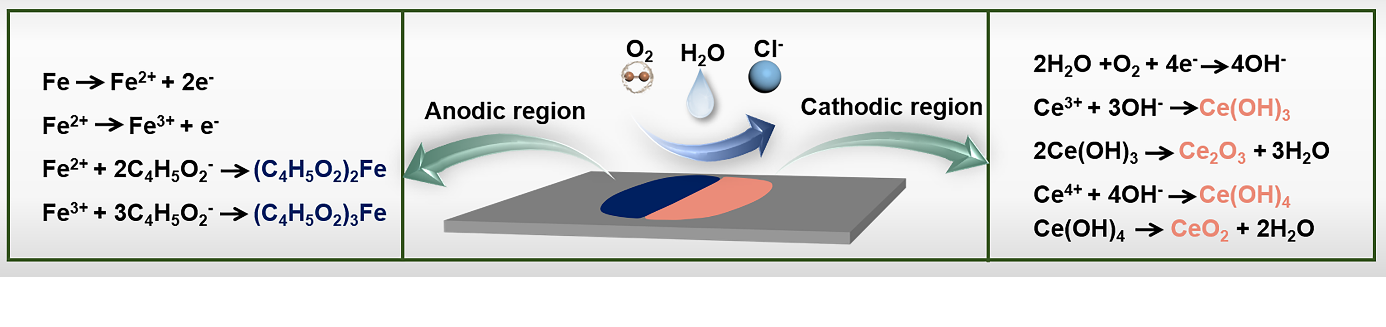
_**

**Fig. S8** Schematic diagram for the formation of passivation layer

**Table S3** Comparison of the comprehensive performance of F-CB/CEP coating with other coatings reported in literature

| TC (W/(m·K)) | Anti-corrosion  performance | *K*_loss_  (%) | WCA  (°) | OCA  (°) | Reference |
| --- | --- | --- | --- | --- | --- |
| 1.63 | 1.1E+11 (150 d) | 3.11 | / | / | [S10] |
| 0.45 | about 1E+04 (120 h) | 10.41 | / | / | [S18] |
| 0.78 | 1.6E+09 (30 d) | 6.28 | / | / | [S19] |
| 0.8 |  | 6.13 | 158 | / | [S20] |
| 0.53 | 1.3E+08 (12 d) | 8.98 | / | / | [S21] |
| 0.434 | complete coating has no corrosion in salt spray test for 144 hours | 10.75 | / | / | [S22] |
| 4.29 | 5.1E+11 (181 d) | 1.20 | 148.35 | 121.67 | F-CB/CEP |

**Note:** Unless otherwise emphasized, the anticorrosion effect of the coating is measured by the |Z|_0.01 Hz_ value (in ohm·cm^2^) after soaking for a period of time. The test time for |Z|_0.01 Hz_ value of different coatings are noted in parentheses. *K*_loss_ is calculated based on the content in Text S11.

**Text S14 Temperature resistance of the F-CB/CEP coating**


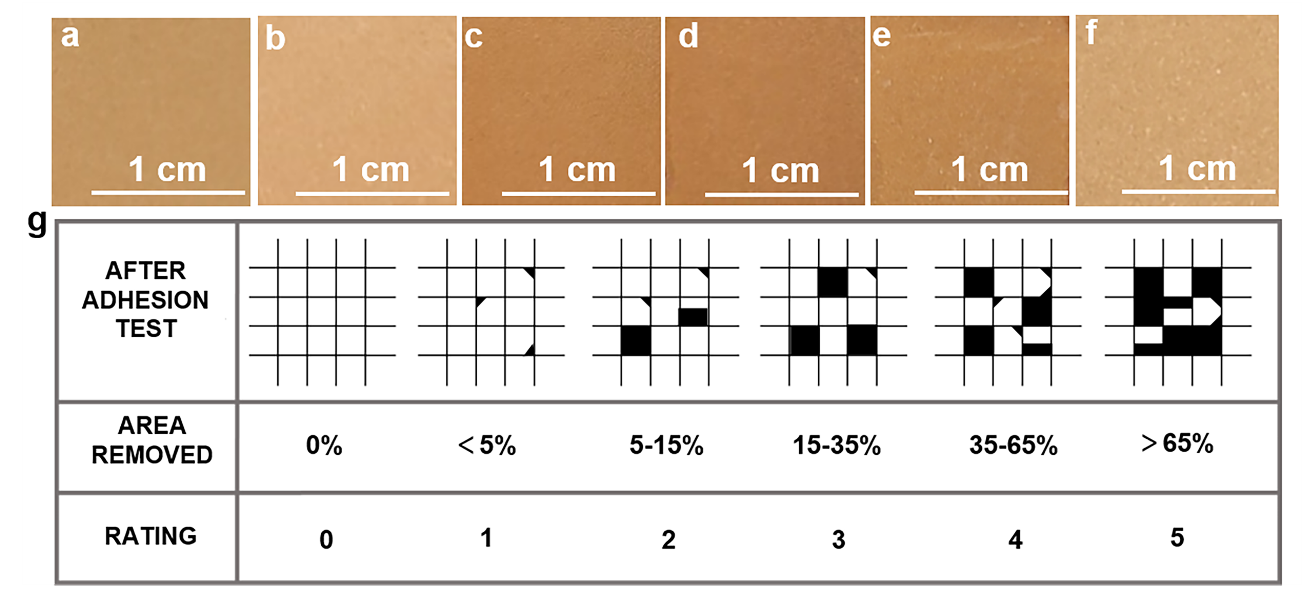


**Fig. S9** The optical photographs of **a** the original F-CB/CEP coating (to avoid repetition, only one photograph of the original coating is present), and the F-CB/CEP coating after **b** 10 air heating cycles at 180 ^o^C, and 10 soak heating cycle**s** at 140 ^o^C in **c** deionized water or solution at pH of **d** 5 or **e** 9, and **f** being placed in constant temperature (50 ^o^C) and humidity (95%) environment for 200 h. **g** The standard in GB/T9286

The temperature resistance of the F-CB/CEP coating was tested. On one hand, the coating was placed in a blast drying oven at 180 °C for 12 h, then cooled to room temperature for 12 h, which is recorded as an air heating cycle. On the other hand, the coating was soaked in reaction kettle filled with deionized water or solution at pH of 5 or 9, respectively. The reaction kettle was placed in a blast drying oven at 140 °C for 10 h, and then cooled to room temperature for 14 h, which is recorded as a soak heating cycle. Optical photographs of the coatings surface were recorded after 10 heating circles and shown in the Fig. S9a-e. It can be clearly seen that even after 10 air heating or soak heating cycles at various high-temperature environment, the coating does not appear to have any layering, wrinkling, bubbling, and cracking. These indicates that the coating has well temperature resistance.

**Text S15 Adhesion of the F-CB/CEP coating**

The adhesion of the F-CB/CEP coating was estimated by the tape-peel test. Coating was scratched by dedicated crosshatch blade with fixed spacing to produce grids with area of 2 mm × 2 mm and the cutting was required to reach the substrate surface. Afterwards, the surface was cleaned by a soft brush, and tape with high stickiness (VHB, 3 M) was tightly pressed on the cut coating surface and was then peeled off vertically and quickly. Repeat this process until the surface of the tape was essentially free of residues of the coating. The results are shown in Fig. 5f and g. It can be seen that after the adhesion test, there is no obvious peeling of the coating and the removed area of the coating is less than 5%, corresponding to the grade 1 adhesion according to the standard in GB/T9286 (Fig. S9g). Notably, the adhesion of the coating after soaking in 3.5 wt% NaCl solution for 302 days still reaches grade 1. These reveal a reliable adhesion between the F-CB/CEP coating and the substrate. This is closely related to the physical and chemical bonds between the coating and the rough substrate surface provided by the epoxy matrix and h-CMAs in the coating [S14, S23, S24]. Moreover, as shown in Fig. S9c-f, even when soaked in various solution at 140 °C for 10 soak heating cycles or placed in constant temperature (50 ^o^C) and humidity (95%) environment for 200 h, the coating does not appear to peel, wrinkle, bubble or crack. It shows the good application prospect of our coating.

**Supplementary References**

1. Y. Zou, Y. Wang, S. Xu, T. Jin, D. Wei, J. Ouyang, D. Jia, Y. Zhou. Superhydrophobic double-layer coating for efficient heat dissipation and corrosion protection. Chem. Eng. J. **362**, 638-649 (2019). https://doi.org/10.1016/j.cej.2019.01.086
2. W.-M. Lu, Z.-P. Shao, J.-B. Hu, X.-Y. Luo, N. Dong, J.-M. Gu. Synthesis, spectra and crystal structure of bis(1,10-phenanthroline)di(μ-α-methylacrylato) lanthanide(iii)dimers. J. Coord. Chem. **40**(1-2), 145-155 (2006). https://doi.org/10.1080/00958979608022853
3. S. Herbers, D. A. Obenchain, K. G. Lengsfeld, H. Kuper, J. A. Becker, J.-U. Grabow. Thermal self polymerization investigated by microwave molecular spectroscopy – rotational characterization of the methyl methacrylate dimer. J. Mol. Spectrosc. **351**, 49-54 (2018). https://doi.org/10.1016/j.jms.2018.07.007
4. H. Qian, M. Zhu, H. Song, H. Wang, Z. Liu, C. Wang. Anti-scaling of superhydrophobic poly(vinylidene fluoride) composite coating: Tackling effect of carbon nanotubes. Progress in Organic Coatings. **142**, 105566 (2020). https://doi.org/10.1016/j.porgcoat.2020.105566
5. C. Xu, Z. Zheng, W. Wu, Z. Wang, L. Fu. Dynamically vulcanized pp/epdm blends with balanced stiffness and toughness via in-situ compatibilization of maa and excess zno nanoparticles: Preparation, structure and properties. Composites, Part B. **160**, 147-157 (2019). https://doi.org/10.1016/j.compositesb.2018.10.014
6. A. Sarnecki, P. Adamski, A. Albrecht, A. Komorowska, M. Nadziejko, D. Moszyński. Xps study of cobalt-ceria catalysts for ammonia synthesis – the reduction process. Vacuum. **155**, 434-438 (2018). https://doi.org/10.1016/j.vacuum.2018.06.034
7. P. Burroughs, A. Hamnett, A. F. Orchard, G. Thornton. Satellite structure in the x-ray photoelectron spectra of some binary and mixed oxides of lanthanum and cerium. J.C.S. Dalton. 1686-1698 (1976). https://doi.org/10.1039/DT9760001686
8. Z. Yuan, W. Li, C. Li, L. Ye. Construction of multiple crosslinking networks in epdm rubber: Synergistic reinforcing effect of graphene-zinc dimethacrylate on epdm and improvement mechanism of sealing resilience. Compos. Part A Appl. Sci. Manuf. **121**, 254-264 (2019). https://doi.org/10.1016/j.compositesa.2019.03.039
9. B. Guo, Y. Lei, F. Chen, X. Liu, M. Du, D. Jia. Styrene–butadiene rubber/halloysite nanotubes nanocomposites modified by methacrylic acid. Appl. Surf. Sci. **255**(5), 2715-2722 (2008). https://doi.org/10.1016/j.apsusc.2008.07.188
10. F. Xu, M. Zhang, Y. Cui, D. Bao, J. Peng, Y. Gao, D. Lin, H. Geng, Y. Zhu, H. Wang. A novel polymer composite coating with high thermal conductivity and unique anti-corrosion performance. Chem. Eng. J. **439**, 135660 (2022). https://doi.org/10.1016/j.cej.2022.135660
11. C. Chai, G. Zhang. Chemical fluid flow and heat transfer, 2nd edn. (Chemical Industry Press; Beijing, China, 2007), pp. 261-275
12. X. Zhang, H. Wang, X. Zhang, Z. Zhao, Y. Zhu. A multifunctional super-hydrophobic coating based on pda modified mos2 with anti-corrosion and wear resistance. Colloid. Surface. A. **568**, 239-247 (2019). https://doi.org/10.1016/j.colsurfa.2019.02.016
13. S. W. Deng, L. B. Tong, X. J. Li, J. H. Chu, M. M. Li, D. N. Zou. Enhanced anti-corrosion/wear properties of mg alloy through a hierarchical bio-inspired self-healing composite coating. Colloid. Surface. A. **658**, 130770 (2023). https://doi.org/10.1016/j.colsurfa.2022.130770
14. M. J. Palimi, E. Alibakhshi, G. Bahlakeh, B. Ramezanzadeh, M. Mahdavian. Electrochemical investigations of the corrosion protection properties of an epoxy-ester coating filled with cerium acetyl acetonate anticorrosive pigment. J. Electrochem. Soc. **164**(13), C709-C716 (2017). https://doi.org/10.1149/2.0231713jes
15. N. Parhizkar, T. Shahrabi, B. Ramezanzadeh. Steel surface pre-treated by an advance and eco-friendly cerium oxide nanofilm modified by graphene oxide nanosheets; electrochemical and adhesion measurements. J. Alloys Compd. **747**, 109-123 (2018). https://doi.org/10.1016/j.jallcom.2018.03.022
16. R. V. Lakshmi, S. T. Aruna, C. Anandan, P. Bera, S. Sampath. Eis and xps studies on the self-healing properties of ce-modified silica-alumina hybrid coatings: Evidence for ce(iii) migration. Surf. Coat. Tech. **309**, 363-370 (2017). https://doi.org/10.1016/j.surfcoat.2016.11.051
17. H. Shi, E.-H. Han, S. V. Lamaka, M. L. Zheludkevich, F. Liu, M. G. S. Ferreira. Cerium cinnamate as an environmentally benign inhibitor pigment for epoxy coatings on aa 2024-t3. Prog. Org. Coat. **77**(4), 765-773 (2014). https://doi.org/10.1016/j.porgcoat.2014.01.003
18. D. Pan, X. Zhang, G. Yang, Y. Shang, F. Su, Q. Hu, R. R. Patil, H. Liu, C. Liu, Z. Guo. Thermally conductive anticorrosive epoxy nanocomposites with tannic acid-modified boron nitride nanosheets. Ind. Eng. Chem. Res. **59**(46), 20371-20381 (2020). https://doi.org/10.1021/acs.iecr.0c04510
19. J. Ding, H. Zhao, H. Yu. Bio-inspired multifunctional graphene-epoxy anticorrosion coatings by low-defect engineered graphene. ACS Nano. **16**, 710-720 (2022). https://doi.org/10.1021/acsnano.1c08228
20. G. G. G. W. K. Kotuwegedara, R. M. de Silva, K. M. N. de Silva, D. P. Dissanayake, L. H. R. Perera, N. P. W. Rathuwadu. Superhydrophobic coating to mitigate metal corrosion with electrically insulative and thermally conductive properties. Mater. Lett. **324**, 132773 (2022). https://doi.org/10.1016/j.matlet.2022.132773
21. D. Zhao, Y. Wang, Y. Chen, P. Zhang, Z. Fu, Q. Yu, H. Huang, J. Shao, Z. Pang, Y. Peng, X. Shen, Y. Zhu, H. Qian, B. Han. Preparation of thermal conductive anticorrosive composite coatings via synergistic effect of carbon nanofillers and heat transfer oil. Colloid Polymer, Sci. **299**(5), 899-908 (2021). https://doi.org/10.1007/s00396-021-04812-x
22. E. Kang, S. Choi, C. Choi, S. E. Shim. Aqueous dispersion of submicron-sized diamond particles for thermally conductive polyurethane coating. Colloid. Surface. A. **415**, 255-261 (2012). https://doi.org/10.1016/j.colsurfa.2012.09.028
23. M. Ramezanzadeh, G. Bahlakeh, B. Ramezanzadeh. Development of a nanostructured ce(iii)-pr(iii) film for excellently corrosion resistance improvement of epoxy/polyamide coating on carbon steel. J. Alloys Compd. **792**, 375-388 (2019). https://doi.org/10.1016/j.jallcom.2019.04.051
24. S. Roshan, A. A. Sarabi. Improved performance of ti-based conversion coating in the presence of ce/co ions: Surface characterization, electrochemical and adhesion study. Surf. Coat. Tech. **410**, 126931 (2021). https://doi.org/10.1016/j.surfcoat.2021.126931
